# COLD REGULATED GENE 27 and 28 antagonize the transcriptional activity of the RVE8/LNK1/LNK2 circadian complex

**DOI:** 10.1093/plphys/kiad210

**Published:** 2023-04-05

**Authors:** Maria L Sorkin, Shin-Cheng Tzeng, Stefanie King, Andrés Romanowski, Nikolai Kahle, Rebecca Bindbeutel, Andreas Hiltbrunner, Marcelo J Yanovsky, Bradley S Evans, Dmitri A Nusinow

**Affiliations:** Donald Danforth Plant Science Center, St. Louis, MO 63132, USA; Division of Biology and Biomedical Sciences, Washington University in St. Louis, St. Louis, MO 63130, USA; Donald Danforth Plant Science Center, St. Louis, MO 63132, USA; Donald Danforth Plant Science Center, St. Louis, MO 63132, USA; Division of Biology and Biomedical Sciences, Washington University in St. Louis, St. Louis, MO 63130, USA; Fundación Instituto Leloir, Instituto de Investigaciones Bioquímicas de Buenos Aires–Consejo Nacional de Investigaciones Científicas y Técnicas (CONICET), Buenos Aires C1405BWE, Argentina; Plant-Environment Signaling Group, Department of Biology, Faculty of Science, Utrecht University, 3584 CH Urecht, The Netherlands; Institute of Biology II, Faculty of Biology, University of Freiburg, Freiburg 79104, Germany; Donald Danforth Plant Science Center, St. Louis, MO 63132, USA; Institute of Biology II, Faculty of Biology, University of Freiburg, Freiburg 79104, Germany; Signalling Research Centres BIOSS and CIBSS, University of Freiburg, Freiburg 79104, Germany; Fundación Instituto Leloir, Instituto de Investigaciones Bioquímicas de Buenos Aires–Consejo Nacional de Investigaciones Científicas y Técnicas (CONICET), Buenos Aires C1405BWE, Argentina; Donald Danforth Plant Science Center, St. Louis, MO 63132, USA; Donald Danforth Plant Science Center, St. Louis, MO 63132, USA

## Abstract

Many molecular and physiological processes in plants occur at a specific time of day. These daily rhythms are coordinated in part by the circadian clock, a timekeeper that uses daylength and temperature to maintain rhythms of ∼24 h in various clock-regulated phenotypes. The circadian MYB-like transcription factor REVEILLE 8 (RVE8) interacts with its transcriptional coactivators NIGHT LIGHT-INDUCIBLE AND CLOCK-REGULATED 1 (LNK1) and LNK2 to promote the expression of evening-phased clock genes and cold tolerance factors. While genetic approaches have commonly been used to discover connections within the clock and between clock elements and other pathways, here, we used affinity purification coupled with mass spectrometry (APMS) to identify time-of-day–specific protein interactors of the RVE8-LNK1/LNK2 complex in Arabidopsis (*Arabidopsis thaliana*). Among the interactors of RVE8/LNK1/LNK2 were COLD-REGULATED GENE 27 (COR27) and COR28, which coprecipitated in an evening-specific manner. In addition to COR27 and COR28, we found an enrichment of temperature-related interactors that led us to establish a previously uncharacterized role for LNK1 and LNK2 in temperature entrainment of the clock. We established that RVE8, LNK1, and either COR27 or COR28 form a tripartite complex in yeast (*Saccharomyces cerevisiae*) and that the effect of this interaction *in planta* serves to antagonize transcriptional activation of RVE8 target genes, potentially through mediating RVE8 protein degradation in the evening. Together, these results illustrate how a proteomic approach can be used to identify time-of-day–specific protein interactions. Discovery of the RVE8-LNK-COR protein complex indicates a previously unknown regulatory mechanism for circadian and temperature signaling pathways.

## Teaser

COLD REGULATED GENE 27 and 28 bind to and antagonize the ability of the circadian clock–associated RVE8/LNK complex to induce target gene expression in the evening in *Arabidopsis thaliana*.

## Introduction

Daily and seasonal patterns in daylength and temperature cycles are 2 of the most dependable environmental cues organisms experience. As such, lifeforms in every kingdom have evolved a mechanism to anticipate and synchronize their biology with the earth's predictable 24 h and 365 d cycles (Ouyang et al. 1998; Rosbash 2009; Edgar et al. 2012). This mechanism is called the circadian clock, which in plants consists of ∼20–30 genes that participate in transcription–translation feedback loops to produce rhythms with a period of about 24 h (Creux and Harmer 2019). These core oscillator genes respond to the environment by producing a physiological response appropriate for a particular time of day or year (Webb et al. 2019). In plants, the clock regulates a variety of phenotypic outputs, including the transition from vegetative to reproductive growth, biotic defense responses, and protection from abiotic stressors such as extreme warm or cold temperature (Greenham and McClung 2015).

Identification of circadian-associated genes has been critical in understanding the generation of biological rhythms. Core oscillator components often exhibit rhythmic gene expression with a period of ∼24 h and a set phase—or time of peak and trough expression. For example, 2 of the first genes to be defined as core oscillator components in the model plant *Arabidopsis thaliana* (Arabidopsis) are the morning-phased MYB-like transcription factors *CIRCADIAN CLOCK ASSOCIATED 1* (*CCA1*) and *LATE ELONGATED HYPOCOTYL* (*LHY*) (Schaffer et al. 1998; Wang and Tobin 1998; Green and Tobin 1999). These genes are highly expressed at dawn and repress the expression of the afternoon- and evening-phased *PSEUDO RESPONSE REGULATOR* genes *PRR1/TIMING OF CAB EXPRESSION 1* (*TOC1*), *PRR5*, *PRR7*, and *PRR9* (Alabadí et al. 2001; Farré et al. 2005; Kamioka et al. 2016). The *PRRs* reciprocally repress *CCA1*/*LHY*, completing one of the negative feedback loops that define the clock. In the evening, *EARLY FLOWERING 3* (*ELF3*), *ELF4*, and *LUX ARRHYTHMO* (*LUX*) interact in the nucleus to form a tripartite protein complex called the evening complex, which represses *PRR9*, *CCA1*/*LHY*, and other clock and growth-promoting factors (Dixon et al. 2011; Nusinow et al. 2011; Chow et al. 2012; Herrero et al. 2012). As we discover additional connections within and between the clock, we enhance our understanding of this important system.

In this study, we used APMS to identify protein–protein interactions associated with the REVEILLE 8 (RVE8)-NIGHT LIGHT-INDUCIBLE AND CLOCK-REGULATED 1 (LNK1)/LNK2 circadian transcriptional complex. The RVEs are an 8-member family of CCA1/LHY-like transcription factors of which some members interact with the LNK proteins to coregulate target gene expression (Rawat et al. 2011; Rugnone et al. 2013; Xie et al. 2014; Pérez-García et al. 2015; Gray et al. 2017). In the late morning, the RVE8-LNK1/LNK2 transcriptional complex activates the expression of evening-expressed clock genes such as *TOC1* and *PRR5* via recruitment of the basal transcriptional machinery to these and other *RVE8* target promoters (Xie et al. 2014; Ma et al. 2018). Conversely, *LNK1/LNK2* are also known to act as corepressors of other *RVE8* targets, such as the anthocyanin structural gene *UDP-GLUCOSE:FLAVONOID 3-O-GLUCOSYLTRANSFERASE (UF3GT)* (Pérez-García et al. 2015). Additionally, LNK1 and LNK2 interact with another transcription factor, MYB3, as corepressors to inhibit the expression of the phenylpropanoid biosynthesis gene *C4H* (Zhou et al. 2017). The mechanism behind the corepressive function of the LNKs and how they switch between an activating and a repressive role is unknown.

LNK1 and LNK2 bind to RVE8 and MYB3 via 2 conserved arginine/asparagine-containing motifs called R1/R2 located in the LNK *C*-terminus (Xie et al. 2014; Zhou et al. 2017). Additionally, the Extra *N*-terminal Tail (ENT) domain present in LNK1 and LNK2 is required for their repressive activity with MYB3 (Zhou et al. 2017). The LNKs have no other known functional protein domains apart from these regions. RVE8 and the other RVEs are characterized by the presence of a LHY-/CCA1-LIKE (LCL) domain, which can directly bind the LNKs, presumably at the *C*-terminus (de Leone et al. 2018; Ma et al. 2018). RVE8 target gene promoters frequently contain the canonical *CCA1*/*LHY*-binding motif called the evening element (EE) as well as G-box-like and morning element (ME)-like motifs (Hsu et al. 2013).

In addition to regulating circadian rhythms, *RVE4*/8 regulate thermotolerance under both high and low temperatures (Li et al. 2019; Kidokoro et al. 2021). After exposure to heat shock, RVE4/8 upregulate the expression of *ETHYLENE RESPONSIVE FACTOR 53* (*ERF53*) and *ERF54*, boosting the plant's heat shock tolerance (Li et al. 2019). In another study, the authors found that *RVE4*/8 also appear to promote freezing tolerance via activation of *DEHYDRATION-RESPONSIVE ELEMENT BINDING PROTEIN 1A* (*DREB1A*, also referred to as *C-REPEAT BINDING FACTOR 3, CBF3*) when grown at 4 °C (Kidokoro et al. 2021). A corresponding association between temperature and the LNKs has not been well studied, although EC-mediated induction of *LNK1* expression under warm nights suggests a role for the LNKs in temperature responses (Mizuno et al. 2014).

Time-of-day is an important variable to consider when studying the activity of most proteins, but especially when considering circadian clock proteins. The daily and seasonal cycling of circadian clock gene expression and other endogenous and external stimuli has prompted researchers to consider how these rhythms influence a given process over the course of the day. RVE8, LNK1, and LNK2 exhibit peak mRNA abundance in the morning, but some level of expression continues throughout the daytime hours (Mockler et al. 2007; Rawat et al. 2011; Rugnone et al. 2013; Xie et al. 2014; Pérez-García et al. 2015). Additionally, previous work has demonstrated time-of-day–specific activity for RVE8 and LNK1/LNK2 (Pérez-García et al. 2015). To better understand the function of these core circadian clock proteins, in this study, we used APMS to identify protein–protein interactions at 2 different times of day: ZT5 and ZT9. Using this approach, we identified time-of-day–specific protein interactions for this circadian transcriptional complex. Temperature response–related GO terms were significantly enriched among the coprecipitated proteins, prompting us to explore and establish a role for LNK1/LNK2 in temperature entrainment of the clock. Among these temperature-related coprecipitated proteins were COLD-REGULATED GENE 27 (COR27) and COR28, which only coprecipitated with RVE8/LNK1/LNK2 at ZT9. Further investigation into the role of the RVE8-LNK1/LNK2-COR27/COR28 interaction suggested that the CORs antagonize activation of RVE8 target genes via the regulation of RVE8 protein stability in the evening. Thus, by taking a proteomic approach to study a core circadian transcriptional complex, we identified an evening-phased RVE8-LNK-COR protein complex that presents an uncharacterized regulatory mechanism for circadian and temperature signaling pathways.

## Results

### Characterization of affinity-tagged lines

**Figure 1.**
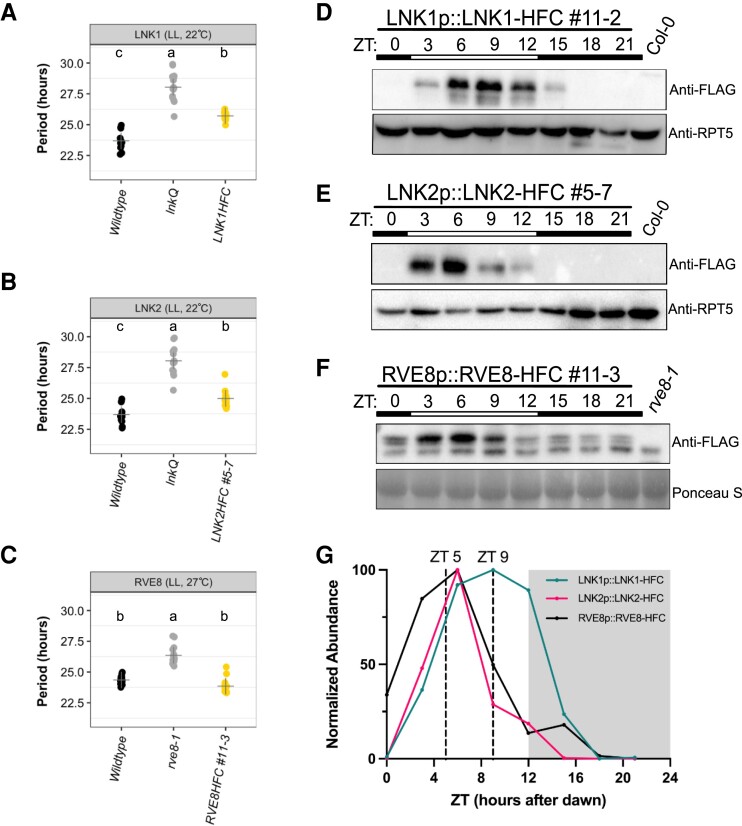
Characterization of affinity-tagged lines used for APMS. **A to C)** Circadian luciferase reporter period analysis of selected T3 homozygous lines expressing **A)** LNK1-HFC, **B)** LNK2-HFC, or **C)** RVE8-HFC in their respective mutant backgrounds (*rve8-1* or *lnkQ*). Each point represents the circadian period of an individual plant, and the + symbol shows the average period for that genotype. Letters correspond to significantly different periods as determined by ANOVA with Tukey's post hoc test. LNK1 and LNK2 luciferase assays were performed together and include the same wild-type and *lnkQ* data. Environmental conditions during imaging are included at the top of the plot (LL, constant light). **D to F)** Time course western blots showing cyclic protein abundance patterns of 10-d-old affinity-tagged lines under 12 h light:12 h dark 22 °C conditions. Affinity-tagged lines are detected with anti-FLAG antibody. RPT5 or Ponceau S staining was used to show loading. Col-0 *CCA1::LUC* (Col-0) or *rve8-1* CCR2::LUC (*rve8-1*) were used as negative controls. White and black bars indicate lights-on and lights-off, respectively. **G)** Twenty-four h protein expression patterns of affinity-tagged lines normalized to Ponceau S or RPT5 quantified by densitometry of western blots shown in **D to F)**. Vertical dotted lines indicate time of tissue collection for APMS. White and gray shading indicates lights-on and lights-off, respectively. Western blots and luciferase reporter assays were repeated at least 2 times. ZT, Zeitgeber time.

To identify interactions with known clock proteins, we created endogenous promoter-driven, 3x-FLAG-6x-His-*C*-terminal (HFC) affinity-tagged versions of RVE8, LNK1, and LNK2. RVE8-HFC was transformed into the *rve8-1 CCR2::LUC* mutant background, while LNK1-HFC and LNK2-HFC were introduced into *lnk1/2/3/4* quadruple mutant (*lnkQ)* (de Leone et al. 2018) *CCA1::LUC.* By transforming our tagged LNKs into the *lnkQ* background, we could eliminate co-precipitating interactors that could be formed through a complex between our tagged LNKs and the endogenous LNKs. To ensure the tagged versions of our proteins of interest functioned similarly to their native counterparts, we selected T3 homozygous lines that rescued the long period mutant phenotype of *rve8-1* or *lnkQ* mutants (Rawat et al. 2011; Xie et al. 2014) (Fig. 1, A to C). LNK1-HFC nor LNK2-HFC fully restored the circadian period back to wild-type levels, but the lengthened period is consistent with the absence of the other 3 LNKs after the introduction of the tagged LNK into the *lnkQ* quadruple mutant (Xie et al. 2014; de Leone et al. 2018). We also determined that the HFC-tagged proteins exhibit rhythmic protein abundance patterns under 12 h light:12 h dark (LD) conditions, as would be expected for these proteins (Figs. 1, D to G and S1). While mRNA expression for *RVE8*, *LNK1*, and *LNK2* peaks at ZT1, ZT5, and ZT2, respectively, peak protein abundance occurred between ZT3-9, ZT3-9, and ZT3-6—about 2–5 h after peak mRNA expression (Mockler et al. 2007) (Figs. 1, D to G, S1, and S2). This lag in protein abundance after transcription is consistent with previously reported data showing a peak in RVE8-HA abundance 3 to 6 h after dawn (Rawat et al. 2011). These experiments demonstrate that our affinity-tagged clock proteins behaved similarly to the native protein and are functional, making them ideal tools for capturing relevant protein interactions.

### APMS identifies time-of-day–specific interacting partners for RVE8, LNK1, and LNK2

**Figure 2.**
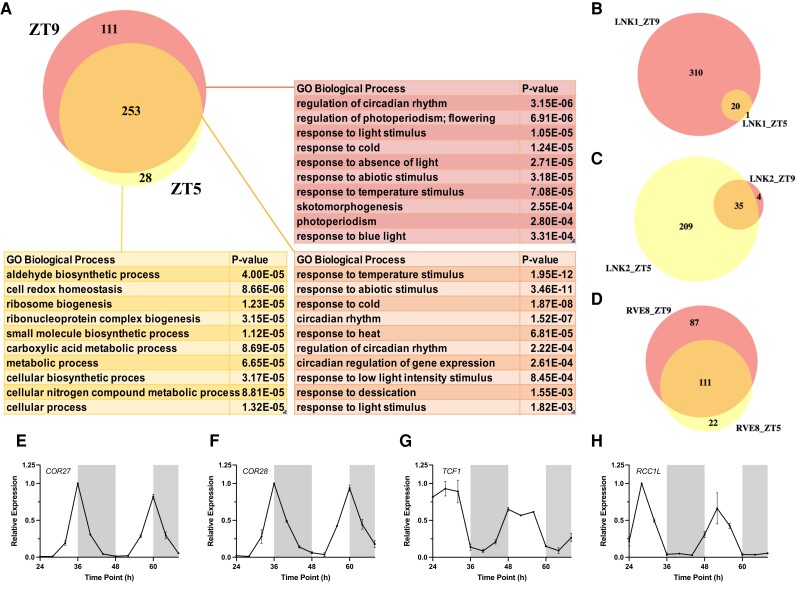
Analysis of proteins coprecipitated with RVE8/LNK1/LNK2-HFC by time-of-day APMS. **A)** Venn diagram showing number of proteins coprecipitated with RVE8/LNK1/LNK2 at ZT5, ZT9, or at both timepoints. Corresponding tables show enriched GO biological process terms with *P*-value. **B to D)** Venn diagrams of coprecipitated proteins at ZT5 and ZT9 separated by bait protein [**B)** LNK1-HFC, **C)** LNK2-HFC, or **D)** RVE8-HFC]. **E to H)** mRNA expression profiles in constant light of 4 cold response proteins identified as RVE8/LNK1/LNK2 interactors. RNA-Seq data for **E to H)** taken from Romanowski et al. (2020)*The Plant Journal*. Error bars represent SEM. ZT, Zeitgeber time.

Based on the 24 h protein expression patterns shown in Figs. 1 and S1, we selected 2 timepoints for APMS: ZT5 and ZT9. We chose these timepoints as they straddle the time of peak expression for these proteins while also providing a 4 h temporal difference to identify time-of-day–specific protein interactions for these clock proteins. We identified a total of 392 proteins that coprecipitated with either RVE8-HFC, LNK1-HFC, or LNK2-HFC at ZT5 or ZT9 but did not coprecipitate in our GFP-HFC nor Col-0 negative controls (Fig. 2A, Supplemental Data Set 1). Consistent with the time of peak LNK1-HFC and LNK2-HFC protein abundance (ZT9 and ZT5, respectively; Fig. 1G), we saw higher total spectra mapping to LNK1-HFC at ZT9 (621) and LNK2-HFC at ZT5 (497) compared to the other timepoint (Tables 1 and 2). Similarly, the number of coprecipitated proteins was greatest at ZT9 for LNK1-HFC and at ZT5 for LNK2-HFC (Fig. 2, B and C, Supplemental Data Set 1). Total spectra mapping to the bait protein RVE8-HFC were similar between the 2 timepoints (Tables 1 and 2). Despite the similarity in RVE8-HFC total spectra between timepoints, we precipitated more ZT9-specific interactors than ZT5-specific interactors with RVE8-HFC (Fig. 2D). Overall, we identified more RVE8/LNK1/LNK2-binding partners at ZT9 (364) versus the earlier timepoint of ZT5 (281) (Fig. 2A) and found that 111 out of 392 (28.3%) total proteins coprecipitated were ZT9-specific; these proteins were not coprecipitated in any APMS experiment performed at ZT5. In summary, the enrichment of coprecipitated proteins at ZT9 suggests an important post-translational role for the RVE8-LNK1/LNK2 complex in the evening.

**Table 1.** Proteins coprecipitated with RVE8/LNK1/LNK2-HFC at ZT5

Protein name	AGI locus number	M.W. (kDa)	LNK1HFC_ZT5_1	LNK1HFC_ZT5_2	LNK2HFC_ZT5_1	LNK2HFC_ZT5_2	RVE8HFC_ZT5_1	RVE8HFC_ZT5_2
LNK1	AT5G64170^a^	70	173	56	0	0	107	87
LNK2	AT3G54500^a^	81	0	0	468	497	154	149
RVE8	AT3G09600^a^	40	22	13	206	223	272	267
RVE6	AT5G52660	36	28	14	79	87	0	0
TCF1	AT3G55580^a^	51	16	8	37	39	7	4
RVE5	AT4G01280^a^	34	16	8	27	33	0	0
RCC1L	AT3G53830^a^	49	4	2	8	8	0	0
RVE4	AT5G02840^a^	31	4	0	85	84	0	0
CCR16	AT1G02150^a^	60	1	0	0	0	0	0
RVE3	AT1G01520^a^	33	0	0	10	11	0	0
GRXS17	AT4G04950	53	0	0	10	9	0	0
UBP12	AT5G06600	131	0	0	8	9	0	0
UBP13	AT3G11910	131	0	0	6	7	1	0
RACK1A	AT1G18080^a^	36	0	0	2	4	0	0
DGR2	AT5G25460^a^	40	0	0	4	3	1	0
PICALM3	AT5G35200^a^	61	0	0	4	2	0	0
TRA1A	AT2G17930	436	0	0	0	2	0	0
CAB4	AT3G47470^a^	28	0	0	1	1	0	0
BTI1	AT4G23630^a^	31	0	0	1	1	0	0
PUB12	AT2G28830^a^	107	0	0	0	1	0	0
ABA1	AT5G67030^a^	74	0	0	0	1	0	0
FLL2	AT1G01320^a^	199	0	0	1	0	0	0
WLIM1	AT1G10200^a^	21	0	0	1	0	0	0
FINS1	AT1G43670^a^	37	0	0	1	0	0	0
PP2A-3	AT2G42500	36	0	0	1	0	0	0
Nucleic acid-binding, OB-fold-like protein	AT3G10090	7	0	0	1	0	0	0
CPNB2	AT3G13470^a^	63	0	0	0	0	31	26
LNK3	AT3G12320^a^	30	0	0	0	0	24	25
SAG24	AT1G66580^a^	25	0	0	0	0	4	0
LNK4	AT5G06980	32	0	0	0	0	3	3
ATRH3	AT5G26742	81	0	0	0	0	1	0

Total spectra for a given coprecipitated protein is shown for each independent ZT5 sample. The curated table excludes coprecipitated proteins that were identified in the GFP-HFC or Col-0 negative control APMS experiments (see Supplemental Data Set 1 for all identifications).

Indicates mRNA is circadian regulated in constant light according to analysis in Romanowski et al. (2020)*The Plant Journal*.

**Table 2.** Identified proteins coprecipitated with RVE8/LNK1/LNK2-HFC at ZT9

Protein name	AGI locus number	M.W. (kDa)	LNK1HFC_ZT9_1	LNK1HFC_ZT9_2	LNK2HFC_ZT9_1	LNK2HFC_ZT9_2	RVE8HFC_ZT9_1	RVE8HFC_ZT9_2
LNK1	AT5G64170^a^	70	435	621	0	0	66	90
LNK2	AT3G54500^a^	81	0	0	140	151	109	137
RVE8	AT3G09600^a^	40	71	101	33	52	285	317
RVE6	AT5G52660	36	86	113	21	22	4	3
RVE5	AT4G01280^a^	34	32	51	12	12	0	0
TCF1	AT3G55580^a^	51	32	49	20	30	13	13
RVE4	AT5G02840^a^	31	31	43	0	7	0	0
RCC1L	AT3G53830^a^	49	20	25	3	10	0	0
COR28	AT4G33980^a^	26	11	15	6	6	26	29
RVE3	AT1G01520^a^	33	8	10	0	0	0	0
CCR16	AT1G02150^a^	60	3	8	0	0	0	1
TRA1A	AT2G17930	436	3	7	0	0	0	0
DGR2	AT5G25460^a^	40	3	4	0	0	2	5
ENTH/ANTH/VHS superfamily protein	AT5G35200^a^	61	2	3	0	0	0	0
FLL2	AT1G01320^a^	199	0	3	0	0	0	1
Nucleic acid-binding, OB-fold-like protein	AT2G40660^a^	42	3	2	0	0	0	0
UBP12	AT5G06600	131	0	1	0	0	0	0
PHOT2	AT5G58140	102	1	2	0	0	0	0
MLK4	AT3G13670^a^	79	0	2	0	0	6	6
UBP13	AT3G11910	131	0	2	0	0	1	4
COR27	AT5G42900	27	2	1	0	1	4	7
WLIM1	AT1G10200^a^	21	1	1	0	0	1	2
CAB4	AT3G47470^a^	28	1	1	0	0	1	1
MLK2	AT3G03940	78	0	0	1	1	6	7
GRXS17	AT4G04950	53	0	0	0	0	1	2
CPNB2	AT3G13470^a^	63	0	0	0	0	35	41
LNK3	AT3G12320^a^	31	0	0	0	0	18	23
LNK4	AT5G06980	32	0	0	0	0	2	6
COP1	AT2G32950^a^	76	0	0	0	0	3	4
MLK1	AT5G18190	77	0	0	0	0	3	4
SPA1	AT2G46340^a^	115	0	0	0	0	1	4
MLK3	AT2G25760	76	0	0	0	0	3	0

Total spectra for a given coprecipitated protein is shown for each independent ZT9 sample. The curated table excludes coprecipitated proteins that were identified in the GFP-HFC or Col-0 negative control APMS experiments (see Supplemental Data Set 1 for all identifications).

Indicates mRNA is circadian regulated in constant light according to analysis in Romanowski et al. (2020)*The Plant Journal*.

We used gene ontology (GO) analysis to categorize coprecipitated proteins at ZT5, ZT9, and ZT5/9 (Fig. 2A). Proteins coprecipitated at ZT5 only were mostly assigned GO biological process terms associated with homeostasis and general metabolism, while proteins found at ZT9 only or ZT5/ZT9 fell into relevant categories such as “regulation of circadian rhythm,” “response to light stimulus,” and “photoperiodism” (Fig. 2A). We also noted that GO terms associated with temperature response were enriched in our interactor data set (“response to cold,” “response to temperature stimulus,” and “response to heat”) (Fig. 2A). This analysis suggested that we identified biologically relevant interacting partners involved in circadian rhythms in our APMS experiments and that there is an enrichment of temperature-related factors among these interactors. We also cross-referenced our lists of coprecipitated proteins with known cycling genes (Romanowski et al. 2020) and found that 71.0% of ZT5% and 71.1% of ZT9 proteins exhibited cyclic mRNA expression (Supplemental Data Set 1), demonstrating that our bait circadian clock proteins mostly interacted with proteins whose expression also cycles.

Among the top interactors for LNK1-HFC, LNK2-HFC, and RVE8-HFC were 4 cold response proteins: COLD-REGULATED GENE 27 (COR27), COR28, and 2 regulators of chromosome condensation family proteins, TOLERANT TO CHILLING/FREEZING 1 (TCF1), and a homolog of TCF1 that we named REGULATOR OF CHROMOSOME CONDENSATION 1-LIKE (*RCC1L*, AT3G53830) (Tables 1 and 2). We characterized these as high-priority interactors based on their previously published roles in environmental signaling (Ji et al. 2015; Li et al. 2016; Wang et al. 2017; Kahle et al. 2020; Li et al. 2020; Zhu et al. 2020), subcellular localization prediction, and mRNA expression patterns. All 4 proteins are predicted to be nuclear localized according to the SUBACon subcellular localization consensus algorithm (Hooper et al. 2014), which stands in agreement with being interactors of the nuclear-localized RVE8/LNK1/LNK2 proteins. Additionally, the mRNA expression for these genes is rhythmic under constant light conditions, suggesting circadian regulation of their expression (Fig. 2, E to H). TCF1 and RCC1L were coprecipitated with RVE8, LNK1, and LNK2 at both ZT5 and ZT9, while COR27 and COR28 were ZT9-specific interactors (Tables 1 and 2).

*TCF1* and *RCC1L* are homologs of the regulator of chromosome condensation (RCC) family protein, RCC1 (Ji et al. 2015), and share 49.7% identity in an amino acid alignment (Supplemental Fig. S3). RCC1 is a highly conserved guanine nucleotide exchange factor (GEF) for the GTP-binding protein RAN and is involved in nucleocytoplasmic export along with the regulation of the cell cycle via chromosome condensation during mitosis (Ren et al. 2020). While there are no previous publications characterizing *RCC1L*, its sister gene *TCF1* is a known negative regulator of cold tolerance in Arabidopsis via the lignin biosynthesis pathway (Ji et al. 2015). *RCC1L* expression is downregulated upon cold treatment (Supplemental Table S1), but no formal studies have been made into its role in cold tolerance nor chromatin biology.

COR27 and COR28 have no known protein domains and are repressors of genes involved in cold tolerance, circadian rhythms, and photomorphogenesis (Li et al. 2016; Wang et al. 2017; Kahle et al. 2020; Li et al. 2020; Zhu et al. 2020). Notably, COR27 and COR28 repress *PRR5*, *TOC1*, and *DREB1A*, the same clock and cold tolerance genes that are activated by RVE8 (Rawat et al. 2011; Kidokoro et al. 2021). Null or knock-down mutants of *cor27*/*cor28* exhibit a long period mutant phenotype, similar to that observed for *lnk* and *rve8* mutants (Rawat et al. 2011; Rugnone et al. 2013; Li et al. 2016). As COR27 and COR28 do not contain a known DNA-binding domain, it is not understood how, mechanistically, these factors alter transcription.

Among the 111 evening-specific interactors were COR27, COR28, CONSTITUTIVELY PHOTOMORPHOGENIC 1 (COP1), and SUPPRESSOR OF PHYA-105 (SPA1) (Table 2). COP1 and SPA1 were RVE8-HFC-specific interactors, while COR27 and COR28 coprecipitated at ZT9 with LNK1-, LNK2-, and RVE8-HFC. We hypothesized that this time-of-day–specific coprecipitation could be explained by the relative abundance of these proteins at ZT5 versus ZT9 due to diurnal changes in gene expression over the course of the day. To investigate this hypothesis, we overlayed the LD mRNA expression patterns of these ZT9-specific interactors on top of the protein abundance levels of RVE8-HFC, LNK1-HFC, and LNK2-HFC that were determined by time course western blots shown in Fig. 1, D to F (Supplemental Fig. S4). There is very little overlap in the expression between *COR27/COR28 and RVE8/LNK1/LNK2* at ZT5 (Supplemental Fig. S4), indicating that COR27/COR28 may have only coprecipitated at ZT9 due to increased expression at that timepoint. In contrast, there was not a clear time-of-day distinction in expression overlap between COP1/SPA1 and the clock bait proteins, suggesting the ZT9-specific interaction between COP1/SPA1 and RVE8-HFC is possibly due to a factor other than expression level, such as recruitment through other proteins (such as COR27 or COR28) (Supplemental Fig. S4).

### COR27 and COR28 interact with circadian and light signaling proteins

To better understand the role of COR27/COR28 at the protein level, we performed APMS using 35S::YFP-COR27 and 35S::GFP-COR28 lines (Li et al. 2016) collected at ZT9. Through this experiment, we validated the interactions between the CORs and RVE8/LNK1/LNK2 and additionally coprecipitated RVE5 and RVE6, further supporting the connection between COR27/COR28 and the RVE/LNK proteins (Table 3, Supplemental Data Set 1). Previous studies have shown an interaction between COR27/COR28 and PHYTOCHROME B (PHYB), COP1, and SPA1 (Kahle et al. 2020; Li et al. 2020; Zhu et al. 2020). Our affinity purification captured these known interactions and additionally identified PHYD, SPA2, SPA3, and SPA4, supporting the previously demonstrated role for COR27 and COR28 in photomorphogenesis (Table 3) (Kahle et al. 2020; Li et al. 2020; Zhu et al. 2020). TCF1, one of the cold tolerance proteins (Ji et al. 2015) to coprecipitate with RVE8/LNK1/LNK2, was also captured with COR27 (Table 3), which further implicates the CORs in freezing tolerance. In total, we identified 268 proteins that coprecipitated with YFP-COR27 or GFP-COR28 (Supplemental Data Set 1). Of these, we found 58.9% exhibited circadian-regulated mRNA (Romanowski et al. 2020). Together, the COR27/COR28 APMS provides strong evidence that these proteins are important factors in circadian and light signaling networks.

**Table 3.** Identified proteins coprecipitated with YFP-COR27/GFP-COR28 at ZT9

Protein name	AGI locus number	M.W. (kDa)	YFP-COR27_ZT9_1	YFP-COR27_ZT9_2	YFP-COR27_ZT9_3	YFP-COR27_ZT9_4	GFP-COR28_ZT9_1	GFP-COR28_ZT9_2	GFP-COR28_ZT9_3	GFP_COR28_ZT9_4
COR27	AT5G42900	27	89	85	95	80	0	0	0	0
COR28	AT4G33980^a^	26	0	0	0	0	22	18	8	10
COP1	AT2G32950^a^	76	16	12	19	16	6	6	1	2
SPA1	AT2G46340^a^	115	16	12	16	13	4	6	1	0
MLK4	AT3G13670^a^	79	16	11	15	12	0	0	0	0
MLK2	AT3G03940	78	13	12	15	12	0	0	0	0
PHYD	AT4G16250	129	8	7	13	11	0	0	0	0
MLK1	AT5G18190	77	10	10	12	10	0	0	0	0
SPA4	AT1G53090	89	8	4	11	7	0	0	0	0
SPA2	AT4G11110	115	8	6	10	6	0	1	0	0
SF1	AT5G51300	87	7	12	7	5	0	0	0	0
RVE8	AT3G09600^a^	40	7	5	7	6	3	4	0	3
LNK2	AT3G54500^a^	81	6	5	6	3	0	1	0	0
MLK3	AT2G25760	76	5	6	6	8	0	0	0	0
LNK1	AT5G64170^a^	70	4	4	5	4	4	3	1	0
SPA3	AT3G15354^a^	93	4	4	4	4	0	0	0	0
RVE6	AT5G52660	36	3	3	4	1	1	0	0	0
RVE5	AT4G01280^a^	34	1	1	2	0	1	0	0	0
CCR2	AT2G21660^a^	17	1	0	1	1	0	0	0	0
TCF1	AT3G55580^a^	51	0	0	1	0	0	0	0	0
PHYE	AT4G18130^a^	123	1	0	0	0	0	0	0	0

Total spectra for a given coprecipitated protein is shown for each independent ZT9 sample. The curated table excludes coprecipitated proteins that were identified in the GFP-HFC or Col-0 negative control APMS experiments (see Supplemental Data Set 1 for all identifications).

Indicates mRNA is circadian regulated in constant light according to analysis in Romanowski et al. (2020)*The Plant Journal*.

### RVE8, LNK1, and COR27/COR28 form a protein complex

**Figure 3.**
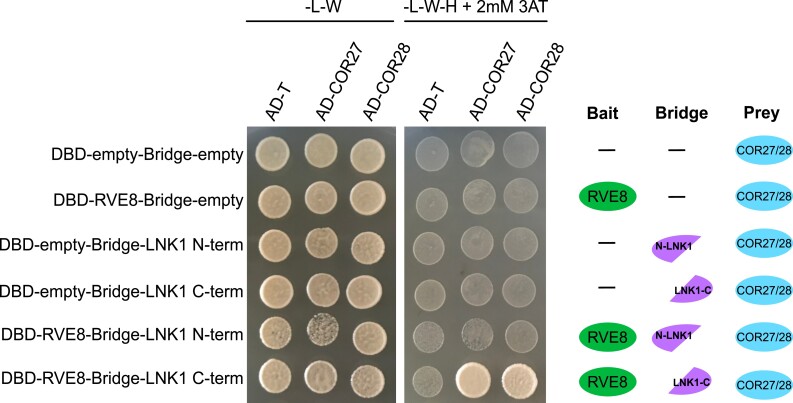
COR27/COR28 interact with RVE8/LNK1 in a yeast 3-hybrid system. Yeast strains Y2H gold or Y187 expressing pBridge (GAL4-DBD and a bridge protein) or pGADT7 (GAL4-Ad), respectively, were mated and plated onto selective media. Successful matings can grow on -Leucine/-Tryptophan media (-L-W), while positive interactors can grow on -Leucine/-Tryptophan/-Histidine + 2 mM 3-amino-1,2,4-triazole (3AT) (-L-W-H + 2 mM 3AT). A graphical depiction of different combinations is shown to the right. Ad-T (large T-antigen protein) is a negative control for prey interactions. Experiment was repeated at least twice.

We used a yeast 2-hybrid system to validate the interactions identified in our APMS between RVE8/LNK1/LNK2 with COR27/COR28. Surprisingly, we did not see a positive interaction between these components when using a binary yeast 2-hybrid (Supplemental Fig. S5**)**. Since APMS can identify both direct and indirect protein–protein interactions, we hypothesized that RVE8-LNK1/LNK2-COR27/COR28 could be forming a protein complex where the CORs can only bind when both RVE8 and LNK1 are present. To test this, we used a yeast 3-hybrid system in which a linker protein is expressed in addition to the bait and prey proteins. We used *N*- and *C*-terminal truncations of LNK1 since full-length LNK1 autoactivates in yeast, as has been shown previously and here (Supplemental Fig. S6) (Xie et al. 2014). Using this method, we found that yeast expressing RVE8, the *C*-terminus of LNK1, and COR27 or COR28 were able to grow on selective media in a higher order complex (Fig. 3). Yeast strains where COR27 or COR28 was paired with either LNK1 or RVE8 alone were unable to grow on selective media, indicating that all 3 components must be present for the CORs to bind (Figs. 3 and S5). We also confirmed that RVE8 interacts with the *C*-terminus of LNK1 (Supplemental Fig. S5), in agreement with previous studies (Xie et al. 2014). In combination with our time-of-day APMS, these results show the CORs interact with RVE8/LNK1 in a complex that is present at ZT9.

### COR27/COR28 alter diurnal RVE8 protein abundance patterns and antagonize activation of the RVE8 target gene *TOC1*

We next sought to determine the biological relevance of the RVE8-LNK1/LNK2-COR27/COR28 interaction. Several recent papers demonstrate that COR27/COR28 interact with and are targeted for degradation by the COP1-SPA1 complex (Kahle et al. 2020; Li et al. 2020; Zhu et al. 2020), which is an E3 ligase complex responsible for targeting circadian and photomorphogenesis factors for degradation by the 26S proteasome (Ponnu and Hoecker 2021). As COR27/COR28 were identified as ZT9-specific RVE8-HFC interactors (Table 2), we hypothesized that COR27/COR28 could recruit an E3 ligase complex like COP1-SPA1 to target RVE8-LNK1/LNK2 for degradation in the evening, thus blocking the expression of RVE8 target genes late in the day. To determine if RVE8-HFC abundance patterns are driven by a post-translational mechanism, we examined protein abundance of RVE8-HFC in seedlings treated with either the 26S proteasome inhibitor bortezomib or DMSO (mock). The mock treated seedlings showed the typical pattern for RVE8-HFC protein abundance with decreasing RVE8-HFC from ZT6 to ZT15 (Supplemental Fig. S7). When treated with bortezomib, RVE8-HFC abundance was stabilized, indicating that the typical decrease in abundance after ZT6 is driven by protein degradation and not a decrease in mRNA abundance (Supplemental Fig. S7).

**Figure 4.**
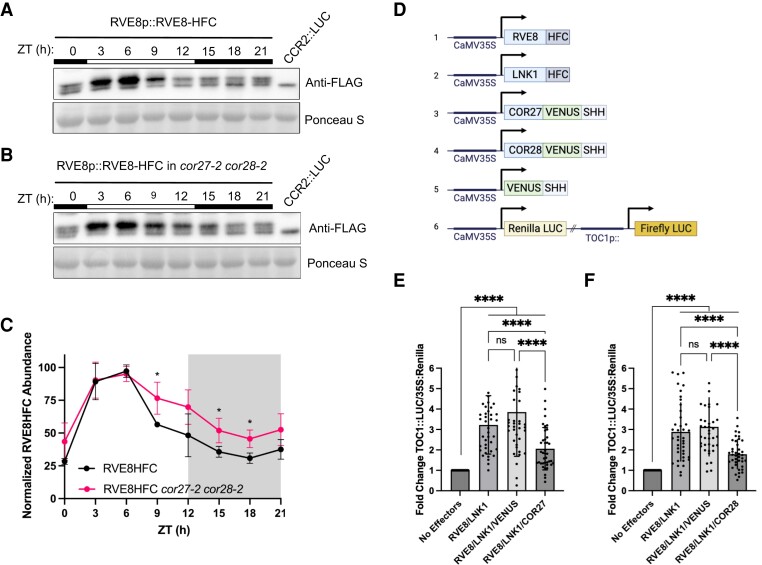
COR27/COR28 alter RVE8-HFC protein abundance patterns and inhibit RVE8/LNK1-mediated activation of *TOC1*. **A to B)** Twenty-four h protein expression patterns of RVE8-HFC in wild-type **A)** or *cor27-2 cor28-2***B)** backgrounds analyzed by western blot. Tissue was collected every 3 h from 12-d-old plants grown under 12 h light:12 h dark 22 °C conditions. Anti-FLAG antibody was used to detect RVE8-HFC, and Ponceau S staining was used to show loading. White and black bars indicate lights-on and lights-off, respectively. Col-0 *CCR2::LUC* was used as the negative control. **C)** Densitometry quantification of **A)** and **B)** RVE8-HFC 24 h abundance normalized to Ponceau S in wild-type and *cor27-2 cor28-2* backgrounds. Points represent the average normalized RVE8-HFC abundance from 3 (WT) or 4 (*cor27-2 cor28-2*) independent bioreps. Asterisks indicate significant differences between genotypes based on Welch’s *t*-test (*P < 0.05). Error bars = Sd. **D)** Schematic of expression constructs used during dual-luciferase assay in *N. benthamiana* (SHH, 2X-StrepII-HA-His_6_ tag; HFC, 6X-His-3X-FLAG *C*-terminus; LUC, luciferase). **E to F)** Luminescence from a dual firefly/renilla luciferase reporter was measured alone (no effectors) or after coinfection with 35S::RVE8-HFC, 35S::LNK1-HFC, 35S::VENUS-SHHc, 35S::COR27-VENUS-SHHc **E)**, or 35S::COR28-VENUS-SHHc **F)**. Luminescence was normalized to constitutively expressed renilla luciferase luminescence to control for infection efficiency and normalized to the no effector sample for that leaf to report the fold change. Points represent the normalized luminescence from 5 independent experiments with *N* = 8. Mean normalized luminescence is indicated by the bar and error = Sd. Asterisks indicate significant differences by unpaired *t*-test with Welch’s correction (ns = not significant, ****P < 0.0001). ZT, Zeitgeber time.

Next, we tested if COR27 and COR28 regulate RVE8 protein abundance by examining cyclic protein abundance in RVE8p::RVE8-HFC versus RVE8p::RVE8-HFC in *cor27-2 cor28-2*. While RVE8-HFC abundance in the wild-type background exhibits rhythmic protein abundance with peak protein levels at ZT6, RVE8-HFC abundance is significantly higher in the *cor27-2 cor28-2* background during the evening and nighttime hours (Fig. 4, A to C and S8). This result is consistent with the hypothesis that in the absence of COR27/COR28, RVE8-HFC should be stabilized specifically in the evening—when it would normally be degraded through its interaction with the CORs. As the circadian rhythm of *RVE8* mRNA expression under LD cycles was shown to be unchanged in the *cor27-2 cor28-2* background (Wang et al. 2017), our results indicate that COR27/COR28 regulate RVE8-HFC protein abundance at the post-translational level. While previous studies have connected COR27/COR28 with COP1-SPA1, the potential involvement of the E3 ligase complex in the degradation of RVE8 has not been explored here and will be of great interest in future work.

We then tested the effect of the CORs on RVE8/LNK1 transcriptional activity using a transactivation assay in *Nicotiana benthamiana* (Fig. 4, D to F). RVE8 binds to the evening element *cis*-regulatory motif in the *TOC1* promoter to activate its expression (Rawat et al. 2011). When LNK1 and RVE8 were transiently expressed together in *N. benthamiana* along with a *TOC1* promoter-driven luciferase reporter, we observed activation of the reporter, as expected (Fig. 4, E and F). When COR27 or COR28 was added to the inoculation cocktail, activation of the reporter was reduced, indicating that the CORs antagonize RVE8/LNK1 transcriptional activity in vivo (Fig. 4, E and F). Taken together, our results indicate that the RVE8-COR27/COR28-LNK1/LNK2 interaction serves to block activation of RVE8 target genes, likely via degradation of RVE8 in the evening.

### The RVEs are important for cold temperature induction of COR27/COR28

*COR27/COR28* contain evening elements in their promoters that are important for their cold induction and could be targets of RVE8 transcriptional regulation (Mikkelsen and Thomashow 2009; Wang et al. 2017). Additionally, *COR27/COR28* are significantly upregulated in an inducible RVE8:GR line according to a previously published RNA-Seq data set (Hsu et al. 2013). Both COR27/COR28 and RVE4/RVE8 regulate cold tolerance in Arabidopsis; *COR27/COR28* expression is induced by cold temperature (16 °C and 4 °C) within 3 h, and the *cor27-1 cor28-2* loss-of-function mutant shows increased freezing tolerance, suggesting these genes are negative regulators of the plant's response to freezing temperatures (Li et al. 2016). In contrast, RVE4 and RVE8 are activators of cold tolerance (Kidokoro et al. 2021). Upon cold treatment (4 °C for 3 h), RVE4 and RVE8 localize to the nucleus and upregulate *DREB1A* to promote freezing tolerance (Kidokoro et al. 2021).

To determine if the RVE transcription factors are regulators of *COR27*/*COR28* cold induction, we examined *COR27*/*COR28* expression at 22 °C and 4 °C in Col-0, *rve8-1*, *rve34568*, and *lnkQ* mutants. We found that *COR27*/*COR28* cold induction was greatly attenuated in *rve34568* and *lnkQ* mutants, consistent with the *CORs* being targets of the RVE-LNK transcriptional complex (Supplemental Fig. S9, A to B). The absence of an effect in the *rve8-1* single mutant suggests there is redundancy among the *RVE* family in the regulation of *COR27/COR28*. Indeed, we found that the LNKs coprecipitated RVE3/4/5/6/8 in our APMS (Tables 1 and 2), suggesting multiple RVE/LNK complexes could influence the regulation of the *CORs*. Interestingly, we saw little effect of RVEs/LNKs on *COR27/COR28* expression at 22 °C at ZT12 (Supplemental Fig. S9, C and D), suggesting these clock factors only have an effect under cold stress or that there may be a greater effect on the expression at 22 °C at a different time of day.

### LNK1 and LNK2 are important for temperature entrainment of the clock

**Figure 5.**
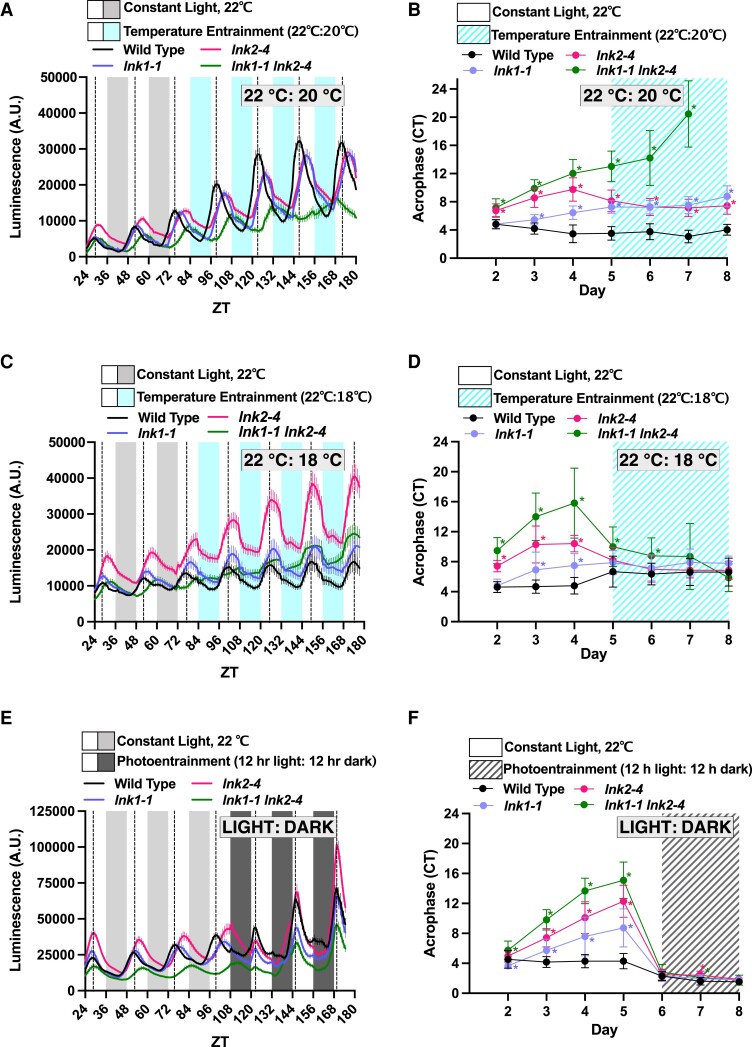
LNK1 and LNK2 are important for temperature entrainment of the clock. **A), C), and E)** Plants were grown for 7 d under 12 h light:12 h dark 22 °C conditions for initial entrainment. On Day 7, seedlings were transferred to imaging chamber and luminescence was measured for at least 3 d in continuous light and temperature (22 °C) before the chamber was switched to either a temperature **A) and C)** or photo **E)** entrainment program. Temperature entrainment consisted of a day temperature of 22 °C and nighttime temperature of 20 °C **A)** or 18 °C **C)**. Photoentrainment consisted of 12 h light followed by 12 h darkness (22 °C). Lines represent the average luminescence from *n* = 16 seedlings with errors bars = SEM. Vertical dotted lines correspond to the acrophase, or time of peak reporter expression, of the *CCA1::LUC* reporter in wild-type plants. **B), D), and F)** Acrophase is plotted for each genotype for each day of imaging in constant light and the temperature entrainment condition **B) and D)** or under photoentrainment **F)**. Each point represents the acrophase of the averaged luminescence trace shown in **A), C), and E)**. Error bars represent standard deviation. Asterisks represent significant differences determined by unpaired *t*-test with Welch’s correction (*P < 0.05). CT, circadian time. A.U., arbitrary units.

The enrichment of temperature response GO terms among the list of coprecipitated proteins in our APMS (Fig. 2A), existing evidence linking RVE8 to temperature regulation (Blair et al. 2019; Kidokoro et al. 2021), and previous work showing overexpression of COR27 or COR28 results in loss of rhythms in temperature cycles (Li et al. 2016) prompted us to investigate whether *LNK1/LNK2* are important for temperature input to the clock. While light is the primary entrainment cue for the plant clock, daily temperature cycles are known to be another major environmental input cue (Devlin and Kay 2001; Salomé and Robertson Mcclung 2005; Avello et al. 2019). To examine temperature entrainment, we examined rhythms from a *CCA1::LUC* reporter in wild-type and *lnk1-1*, *lnk2-4*, and *lnk1-1 lnk2-4* mutant plants that were first grown under constant light and then transferred into a temperature entrainment condition. Under constant light, the *lnk* mutants exhibited their canonical long period mutant phenotype (Rugnone et al. 2013) (Fig. 5, A, C, and E). Upon entering a temperature entrainment condition of 12 h 20 °C:12 h 22 °C, the *lnk1-1 lnk2-4* mutants were unable to resynchronize their circadian rhythms to that of wild type (Fig. 5, A and B). This defect was ameliorated when the difference between the minimum and maximum temperature was increased from 2 °C to 4 °C. When provided temperature cycles of 12 h 18 °C:12 h 22 °C, all mutants were not statistically different from wild-type acrophase (peak reporter expression) by the third day of temperature entrainment (Fig. 5, C to D). However, this resynchronization was still slower than when the *lnk* mutants were provided with photocycles—upon the transition from constant light to LD cycles, all mutants were able to immediately re-align their rhythms to wild type, indicating that the *lnk* mutants are specifically impaired in their ability to use temperature as an entrainment cue (Fig. 5, E and F). These data are also represented as Rayleigh plots, showing the variation in acrophase between lines throughout the experiments (Supplemental Figs. S10–S12).

The temperature entrainment programs used in Fig. 5, A to D, are nonramping, meaning the temperature shifts immediately from the cool to warm temperatures. To better simulate environmental conditions, we also employed a ramping temperature entrainment which gradually oscillated between a low temperature of 16 °C and a high of 22 °C. We observed a similar delay in the ability of the *lnk* mutants to assimilate to wild-type acrophase under ramping temperature cycles, demonstrating that this defect is not a by-product of step temperature changes (Supplemental Fig. S13).

As the LNKs form a 4-member family, we also examined whether LNK3/LNK4 play a role in temperature entrainment. The *lnk3-1 lnk4-1* double mutant showed little difference from wild-type rhythms under constant light nor temperature entrainment, indicating LNK1/LNK2 are the primary family members important for temperature entrainment (Supplemental Fig. S14). In summary, we have demonstrated a previously unknown role for LNK1/LNK2 in temperature entrainment of the clock.

## Discussion

Daily and seasonal temperature cycles are important cues for the entrainment of the plant circadian clock (Salomé and Clung 2005). In parallel to this, the clock is essential for proper response to temperature stimuli (Salomé and Robertson Mcclung 2005; Thines and Harmon 2010). In this study, we have identified a time-of-day–specific interaction between 2 established components of the circadian and temperature response pathways: the circadian clock transcriptional activation complex containing RVE8 and LNK1/LNK2 and the cold response proteins COR27/COR28. Previous studies have demonstrated that RVE8 and COR27/COR28 both regulate the transcription of the master cold response regulator *DREB1A* and the core circadian oscillator genes *PRR5* and *TOC1*; however, RVE8 acts as a transcriptional activator of these targets, while the CORs act as repressors (Rawat et al. 2011; Li et al. 2016; Wang et al. 2017; Kidokoro et al. 2021). In addition to sharing transcriptional targets, RVE8 and COR27/COR28 also affect similar phenotypes, including period lengthening in the null or knock-down mutants and regulation of photoperiodic flowering time (Rawat et al. 2011; Li et al. 2016). Despite these established overlaps in function between the RVE8-LNK1/LNK2 complex and the CORs, a mechanistic connection between these factors has been lacking until now. In this study, we have demonstrated that COR27/COR28 physically interact with and regulate the protein stability of the RVE8-LNK1/LNK2 complex in the evening and that the CORs antagonize RVE8/LNK1-mediated activation of *TOC1* expression.

Our time-of-day–specific APMS experiments demonstrated that RVE8, LNK1, and LNK2 interact with different protein partners at ZT5 versus 4 h later at ZT9 (Fig. 2, A to D). LNK1 and RVE8 interacted with more protein partners at the later timepoint, ZT9, while LNK2 coprecipitated more interactors at ZT5 (Fig. 2, B to D**)**. For LNK1 and LNK2, their time of peak protein abundance (Fig. 1G) aligned with the time of day when they coprecipitated the most interactors (Fig. 2, B and C), suggesting that increased abundance of these clock bait proteins led to an increased number of captured interactions. Interestingly, while our 24 h time course western blots showed a higher abundance of RVE8-HFC at ZT5, we coprecipitated more interactors at ZT9 than at ZT5. This might indicate that even though protein levels of RVE8-HFC are lower at ZT9, perhaps there is an important bridge protein expressed in the evening that links in RVE8-HFC interactors only in the evening. Alternatively, perhaps there are more RVE8-HFC protein-interacting partners expressed at ZT9 than at ZT5. By performing APMS at 2 different timepoints, we have established that these circadian clock proteins interact with different partners depending on the time of day.

For example, COR27, COR28, COP1, and SPA1 were coprecipitated with RVE8/LNK1/LNK2 at ZT9 but not ZT5 (Tables 1 and 2). We have considered the following hypotheses for what is driving this time-of-day–specific interaction: (i) The diurnal expression patterns of these components produces high gene expression overlap at ZT9 but not ZT5, (ii) there is a third protein component that is expressed at ZT9 that allows for the interaction between these factors via bridging or by inducing a conformational change in one of the participating proteins, (iii) a time-of-day–dependent post-translational modification of one of the participating proteins is required for the interaction, or (iv) APMS is not an exclusionary method and could simply have not detected a low abundance peptide that was coprecipitated at ZT5. When we examined the LD mRNA expression patterns for *COR27*, *COR28*, *COP1*, and *SPA1*, we found that COR27 and COR28 are most likely ZT9-specific interactors due to their mRNA expression levels having a higher overlap with RVE8/LNK1/LNK2-HFC protein abundance at ZT9 (Supplemental Fig. S4). Indeed, the *CORs* have very low mRNA expression at ZT5 and thus are likely absent from the cell and not interacting with the RVE8-LNK1/LNK2 proteins (Supplemental Fig. S4). COP1 and SPA1 expression, in contrast, does overlap with RVE8-HFC at ZT9 and ZT5 (Supplemental Fig. S4). We instead think it is possible that COP1/SPA1 could be recruited to RVE8 via COR27/COR28 and thus can only be coprecipitated at ZT9 [hypothesis (ii)]. However, future studies are needed to validate this possibility.

As COR27/COR28 are post-translationally regulated by 26S proteasome-mediated degradation (Kahle et al. 2020; Li et al. 2020; Zhu et al. 2020), we predicted that the interaction between RVE8/LNK1/LNK2 and COR27/COR28 could function to target the circadian transcriptional module for degradation in the evening. We found that RVE8-HFC cyclic protein abundance patterns were disrupted in a *cor27-2 cor28-2* mutant background, with higher RVE8-HFC levels observed specifically during the evening and nighttime hours (Figs. 4, A to C and S8). This suggests that COR27/COR28 are important for degradation of RVE8 in the evening. Furthermore, evening degradation of RVE8-HFC was also reduced by treatment with bortezomib, an inhibitor of the proteasome (Supplemental Fig. S7). As COP1/SPA1 were identified as ZT9-specific RVE8 binding proteins, we suggest that the CORs recruit the COP1-SPA1 E3 ubiquitin ligase complex to RVE8-LNK1/LNK2 to target it for degradation by the proteasome, though this has yet to be directly tested. We also coprecipitated *UBIQUITIN-SPECIFIC PROTEASE 12* (*UBP12*) and *UBP13* and the E3 ubiquitin ligases *PLANT U-BOX 12* (*PUB12*) and *PUB13* in RVE8/LNK1/LNK2 APMS experiments, and these factors may also play a role in time-of-day–specific complex degradation (Tables 1 and 2, Supplemental Data Set 1). In *N. benthamiana* transactivation assays, we observed that the presence of COR27/COR28 reduced the ability of RVE8-LNK1 to activate the expression of a *TOC1* promoter-driven reporter, demonstrating that the CORs have an antagonistic effect on the transcriptional activity of this circadian module (Fig. 4, E and F). This hypothesis predicts that loss of the CORs would increase the stability of the RVE8-LNK1/LNK2 complex, phenocopying a RVE8-OX or LNK-OX plant. However, previous studies have reported that *cor27-2 cor28-2* double mutants exhibit a long period, as do *rve8-1* and *lnkQ* loss-of-function mutants (Rawat et al. 2011; Rugnone et al. 2013; Li et al. 2016; Wang et al. 2017). Furthermore, COR27 and COR28 overexpression also leads to a long circadian period (Li et al. 2016; Wang et al. 2017). While we do not have a clear explanation for these opposing findings, we propose that the high interconnectivity of the circadian clock or genetic compensation could contribute to these confounding period phenotypes.

The CORs do not have identifiable DNA-binding domains and do not bind to DNA in vitro (Li et al. 2020); therefore, the CORs must work with a DNA-binding protein to affect transcription of their target genes. Previous work supported this hypothesis by showing that COR27/COR28 interact with the major photomorphogenic transcription factor ELONGATED HYPOCOTYL 5 (HY5) and regulate some of the same HY5 target loci (Li et al. 2020). Perhaps, a similar mechanism is at work here, with the CORs interacting with the RVE-LNK complex to alter its transcriptional activity. The mechanism behind how the CORs change or potentially change the activity of these transcription factors is an open question.

Finally, as *COR27/COR28* expression is induced under cold stress and RVE8 accumulates in the nucleus upon cold treatment, this presents an interesting possibility that the interaction between RVE8 and the CORs could serve to connect cold temperature response and the circadian clock. Notably, COR27/COR28 and RVE8 oppositely regulate freezing tolerance; the CORs repress the expression of *DREB1A* to decrease freezing tolerance, while RVE4/8 activate *DREB1A* expression (Li et al. 2016; Kidokoro et al. 2021). Thus, we anticipate that the interaction between the CORs and the RVE8-LNK complex is antagonistic in its nature.

**Figure 6.**
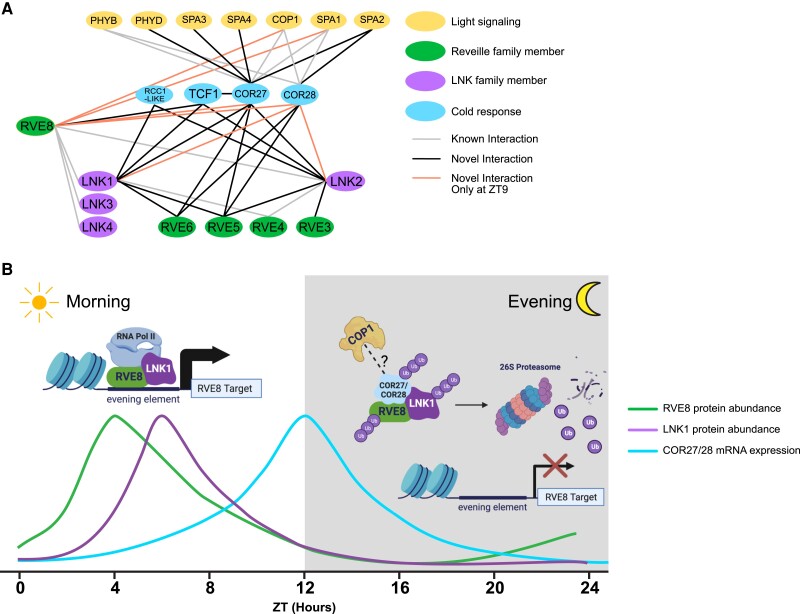
The RVE8-LNK1/LNK2-COR27/COR28 complex is a previously uncharacterized post-translational regulatory mechanism in the circadian clock. **A)** Protein interaction network compiled from APMS experiments using RVE8-HFC, LNK1-HFC, LNK2-HFC, YFP-COR27, and GFP-COR28 as bait proteins at ZT5 and ZT9. Black lines indicate interactions newly identified in this study, gray lines show previously published interactions validated in this study, and orange lines show interactions that were identified only at ZT9. **B)** Model of hypothesized role of the RVE-LNK-COR interaction during a 24 h period. In the morning, RVE8-LNK1/LNK2 interact to coactivate the expression of target genes such as evening-phased circadian clock genes and cold response genes. Toward the evening, COR27/COR28 are expressed and interact with the RVE8-LNK1/LNK2 complex, potentially recruiting a ubiquitin E3 ligase such as COP1 to target the entire complex for degradation by the 26S proteasome, thus blocking activation of RVE8 targets in the evening. Green and purple lines show approximate protein abundance patterns of RVE8 and LNK1, respectively, while the blue line shows approximate *COR27/COR28* mRNA expression.

In summary, we used APMS to identify time-of-day–specific, circadian-associated proteins using the RVE8/LNK1/LNK2 core oscillator proteins as baits. By performing APMS at 2 timepoints during the 24 h cycle, we identified time-of-day–specific interactors, including COR27 and COR28, which only coprecipitated with these 3 clock baits at the later timepoint, ZT9 **(**Fig. 6A and Tables 1 and 2). Taken together, we propose the following model **(**Fig. 6B): In the morning–early afternoon, when the *CORs* are not expressed, the RVE8-LNK1/LNK2 complex is free to perform its canonical duty as an activating force in the circadian oscillator and in cold tolerance. As evening approaches, *COR27*/*COR28* expression rises and the RVE8-LNK1/LNK2-COR27/COR28 complex is formed, which antagonizes RVE8-LNK1/LNK2 transcriptional activity via regulating RVE8 protein abundance. While we think the ZT9-specific interaction between RVE8 and COP1/SPA1 is a good starting point, the specific mechanism by which RVE8 protein abundance is regulated by the 26S proteasome is unknown and will be of interest for future studies. Follow-up work should also examine the effect of RVE8-LNK1/LNK2-COR27/COR28 complex formation on the expression of specific clock and cold tolerance loci at different times of day.

## Materials and methods

### Plant materials

T-DNA disrupted *A. thaliana* lines used in this study: *rve8-1* (SALK_053482C), *lnk1-1* (SALK_024353), *lnk2-1* (GK_484F07), *lnk2-4* (GK_484F07), *lnk3-1* (SALK_085551C), *lnk4-1* (GK_846C06), *cor27-2* (SALK_042072C), and *cor28-2* (SALK_137155C) (Alonso et al. 2003). The *lnkQ CCA1::LUC* line was generated by transforming the *lnkQ* mutant background (de Leone et al. 2020) with a binary vector containing *CCA1::LUC* and Basta resistance (from Harmer Lab). The *lnk3-1 lnk4-1 CCA1::LUC* line was generated by crossing *lnk3-1 lnk4-1* to the *CCA1::LUC* reporter. The 35S::YFP-COR27 and 35S::GFP-COR28 lines were described previously (Li et al. 2016) and generously shared with us by Dr. Hongtao Liu. The *rve8-1 CCR2::LUC* line was described previously (Rawat et al. 2011) and generously shared with us by Dr. Stacey Harmer. The *lnk1-1 CCA1::LUC*, *lnk2-4 CCA1::LUC*, and *lnk1-1 lnk2-4 CCA1::LUC* lines were a generous gift from Dr. Xiaodong Xu (Xie et al. 2014). All plants used were in the Col-0 background.

Seeds were sterilized with chlorine gas for 4–5 h and plated on 1/2X Murashige and Skoog basal salt medium with 0.8% (*w*/*v*) agar + 1% (*w*/*v*) sucrose. After stratification for 2 d, plates were transferred to a Percival incubator (Percival Scientific, Perry, IA) set to a constant temperature of 22 °C. Light entrainment was 12 h light/12 h dark (LD) cycles, with light supplied at 80 *µ*mol/m^2^/s from fluorescent bulbs. Twenty-four h tissue collections were performed under white light during the daytime timepoints and under dim green light during the nighttime timepoints.

### Generation of epitope-tagged lines and plasmid construction

To generate pB7-RVE8-HFC and pB7-RVE8p::RVE8-HFC, RVE8 was cloned from genomic DNA without the stop codon using primers pDAN1127 and pDAN1128 (Supplemental Table S2) and cloned into NotI/AscI-digested pENTR-MCS through In-Fusion HD cloning (Clontech, Mountain View, CA). pENTR-RVE8-no stop was then recombined using LR Clonase (Thermo Fisher Scientific, Waltham, MA) into pB7-HFC (Huang et al. 2016a), which contains the 6X-HIS-3X-FLAG *C*-terminal tag, to generate pB7-RVE8-HFC. To generate the endogenous promoter-driven line, the sequence upstream of the RVE8 transcription start site to the stop codon of the upstream gene was cloned (945 bases) using primers pDAN1129 and pDAN1130 (Supplemental Table S2). The 35S Cauliflower Mosaic Virus (CaMV35S) promoter was excised from pB7-RVE8-HFC via PmeI/SpeI digest and replaced with the RVE8 promoter fragment through In-Fusion HD cloning (Clontech, Mountain View, CA) to generate pB7-RVE8p::RVE8-HFC. pB7-RVE8p::RVE8-HFC binary vector was transformed into *rve8-1 CCR2::LUC* (Rawat et al. 2011) by Agrobacterium (*Agrobacterium tumefaciens*)-mediated transformation, and positive transformants were identified through basta resistance (Clough and Bent 1998). Of 6 homozygous T3 transgenic lines screened, we selected line pB7-RVE8p::RVE8-HFC in *rve8-1 CCR2::LUC* #11-3 as it best rescued the *rve8-1* long period mutant phenotype at 27 °C.

To generate pH7WG2-LNK1p::LNK1-HFC and pH7WG2-LNK2p::LNK2-HFC, LNK1 and LNK2 coding sequences were cloned from cDNA without the stop codon using primers pDAN0990/pDAN0991 (LNK1) and pDAN1066/pDAN1067 (LNK2) (Supplemental Table S2) and recombined into pENTR-MCS through dTOPO cloning or In-Fusion HD cloning (Clontech, Mountain View, CA), respectively. pENTR-LNK1-no stop and pENTR-LNK2-no stop were then recombined using LR Clonase (Thermo Fisher Scientific, Waltham, MA) into pB7-HFC to generate pB7-LNK1-HFC and pB7-LNK2-HFC. To make the endogenous promoter-driven construct, the LNK1 promoter was cloned from the LNK1 transcription start site to the upstream gene's 5′ UTR (1709bp) using primers pDAN1016 and pDAN1017 (Supplemental Table S2). This promoter fragment was swapped with CaMV35S via PmeI/SpeI digest and In-Fusion HD cloning (Clontech, Mountain View, CA) to generate pB7-LNK1p::LNK1-HFC. Of the 13 homozygous T3 transgenic lines screened, we selected line pB7-LNK1p::LNK1-HFC in *lnkQ CCA1:LUC* #11-2 as it best rescued the *lnkQ* long period mutant phenotype at 22 °C. Similarly, the LNK2 promoter was cloned from just before the start of the upstream gene through 142 bases into exon 4 from genomic DNA using primers pDAN1018 and pDAN1019 (Supplemental Table S2) and inserted into pB7-HFC PmeI/BglII digest and In-Fusion HD cloning (Clontech, Mountain View, CA) to generate pB7-LNK2p::LNK2-HFC. To make pH7WG2-LNK1p::LNK1-HFC and pH7WG2-LNK2p::LNK2-HFC, pB7-LNK1p::LNK1-HFC, pB7-LNK2p::LNK2-HFC, and pH7WG2 (Karimi et al. 2002) were digested with KpnI and AgeI and the resulting fragments were ligated. pH7-LNK1p::LNK1-HFC and pH7-LNK2p::LNK2-HFC binary vector were transformed into *lnkQ CCA1::LUC* by agrobacterium mediated transformation and positive transformants were identified through hygromycin resistance (Clough and Bent 1998). Of the 6 homozygous T3 transgenic lines screened, we selected line pB7-LNK2p::LNK2-HFC in *lnkQ CCA1::LUC* #5-7 as it best rescued the *lnkQ* long period mutant phenotype at 22 °C.

To make LNK1 truncations, the *N*-terminus of LNK1 from the start codon through amino acid 296 was cloned using primers pDAN1954/pDAN2010 (Supplemental Table S2), adding a stop codon. The LNK1 *C*-terminal fragment was cloned using primers pDAN2011/pDAN1955 (Supplemental Table S2) with the first amino acid starting at amino acid number 297. Gene fragments were recombined into pENTR-MCS through In-Fusion HD cloning (Clontech, Mountain View, CA) to make pENTR-LNK1-N-term-STOP and pENTR-LNK1-C-term-STOP.

To generate pK7-VENUS-SHHc expression construct, VENUS was cloned from plasmid mVENUS C1 (Koushik et al. 2006) using primers pDAN0868 and pDAN0196 into pENTR-dTOPO to generate pENTR-VENUS. The resulting plasmid was recombined with pK7-SHHc to generate pK7-VENUS-SHHc.

To generate pK7-VENUS (VEN)-2x-StrepII-HA-6X-His-*C*-terminus (SHHc) destination clone, we first made pK7-SHHc by PCR amplifying the 2X-SII-HA-6X-His-*C*-terminal (SHHc) tag from pB7-SHHc (Huang et al. 2016b) and digesting pK7FWG2 (Karimi et al. 2002) with BstXI and KpnI. The PCR fragment containing the SHHc tag was combined with the digested backbone using In-Fusion HD cloning (Clontech, Mountain View, CA) to make pK7-SHHc. VENUS was cloned from plasmid mVENUS C1 (Koushik et al. 2006) using primers pDAN0869 and pDAN0870 and recombined with pK7-SHHc digested with AvrII using In-Fusion HD cloning (Clontech, Mountain View, CA) to generate pK7-VEN-SHHc.

pENTR-no stop clones of COR27 and COR28 were generated by amplifying the coding sequences of COR27 (AT5G24900.1) and COR28 (AT4G33980.1) using primers pDAN1906/pDAN1908, and pDAN1909/pDAN1911, respectively (Supplemental Table S2). The resulting amplicons were cloned into NotI/AscI-digested pENTR-MCS through In-Fusion HD cloning (Clontech, Mountain View, CA) to make pENTR-COR27-no stop and pENTR-COR28-no stop. To generate pK7-COR27-VEN-SHHc, and pK7-COR28-VEN-SHHc, the pENTR-no stop versions of these genes were recombined to the pK7-VEN-SHHc binary vector using LR Clonase (Thermo Fisher). These *C*-terminally tagged proteins are driven from the CaMV35S promoter. To generate the dual-luciferase reporter pGreenII 0800-LUC-TOC1p, 2098 bp of the TOC1 promoter was cloned using primers pDAN2735/pDAN2736 (Supplemental Table S2) and inserted via In-Fusion HD cloning (Clontech, Mountain View, CA) into the pGreenII 0800-LUC plasmid (Hellens et al. 2005) digested with BamHI. The resulting vector (pGreenII 0800-LUC-TOC1p) constitutively expresses renilla luciferase from the CaMV35S promoter and contains the gene for firefly luciferase driven by the TOC1 promoter.

To generate yeast (*Saccharomyces cerevisiae*) 2-/3-hybrid vectors, the gene of interest was cloned from its pENTR-STOP template using primers pDAN2349/pDAN2350 (Supplemental Table S2) and recombined into pGADT7 digested with EcoRI using In-Fusion HD cloning (Clontech, Mountain View, CA). For cloning into pGBKT7, primers pDAN2347/pDAN2348 (Supplemental Table S2) were used to clone off the pENTR-STOP template and recombine into BamHI-digested pGBKT7 using In-Fusion HD cloning (Clontech, Mountain View, CA). For cloning into the pBridge vector (Clontech, Mountain View, CA), the gene of interest was cloned from its pENTR-STOP template using primers pDAN2441/pDAN2442 (Supplemental Table S2) and recombined into the first MCS of pBridge digested with EcoRI using In-Fusion HD cloning (Clontech, Mountain View, CA) or using primers pDAN2443/pDAN2444 (Supplemental Table S2) to recombine into the second MCS of pBridge digested with BglII using In-Fusion HD cloning (Clontech, Mountain View, CA).

All vectors were verified by sequencing before use.

### Affinity purification

Affinity purification was performed as detailed in Sorkin and Nusinow (2022). Briefly, affinity-tagged lines were plated on 1/2x MS + 1% (*w*/*v*) sucrose and grown for 10 d under white light, 12 h light:12 h dark, constant 22 °C conditions. On Day 10 of growth, tissue was harvested at either ZT5 or ZT9 in the light. To extract protein, powdered tissue was resuspended in SII buffer [100 mM sodium phosphate, pH 8.0, 150 mM NaCl, 5 mM EDTA, 5 mM EGTA, 0.1% (*v*/*v*) Triton X-100, 1 mM PMSF, 1x protease inhibitor mixture (Roche, Basel, Switzerland), 1x Phosphatase Inhibitors II & III (Sigma-Aldrich), and 5 *µ* M MG132 (Peptides International, Louisville, KY)] and sonicated using a duty cycle of 20 s (2 s on, 2 s off, total of 40 s) at 50% power. Extracts were clarified of cellular debris through 2× centrifugation for 10 min at ≥20,000 × g at 4 °C.

For HFC-tagged samples, clarified extracts were incubated with FLAG-M2-conjugated Protein G Dynabeads (Thermo Fisher Scientific, Waltham, MA) for 1 h. Captured proteins were eluted off FLAG beads using 500 *µ*g/mL 3x-FLAG peptide (Sigma-Aldrich). Eluted proteins were then incubated with Dynabeads His-Tag Isolation and Pulldown (Thermo Fisher Scientific, Waltham, MA) for 20 min and then washed 5 × 1 min in His-Tag Isolation Buffer (100 mM Na-phosphate, pH 8.0, 150 mM NaCl, 0.025% Triton X-100). Washed bead pellet was washed 4× in 25 mM ammonium bicarbonate and flash frozen in liquid N_2_.

For YFP-COR27 and GFP-COR28, clarified extracts were incubated with GFP-TRAP Magnetic Agarose affinity beads (ChromoTek GmbH, Planegg-Martinsried, Germany) for 1 h. Captured proteins were washed 3 × 1 min in His-Tag Isolation Buffer (100 mM Na-phosphate, pH 8.0, 150 mM NaCl, 0.025% Triton X-100) and 4× in 25 mM ammonium bicarbonate and then flash frozen in liquid N_2_.

### Liquid chromatography–mass spectrometry analysis of AP samples

Samples on affinity beads were resuspended in 50 mM ammonium bicarbonate, reduced (10 mM TCEP), and alkylated (25 mM iodoacetamide) followed by digestion with Tryspin at 37 °C overnight. Digest was separated from beads using a magnetic stand and acidified with 1% TFA (*v*/*v*) before cleaned up with C18 tip (Thermo Fisher Scientific, Waltham, MA). The extracted peptides were dried down, and each sample was resuspended in 10 *µ*L 5% ACN/0.1% FA. Five *µ*L was analyzed by liquid chromatography–mass spectrometry (LC–MS) with a Dionex RSLCnano HPLC coupled to a Orbitrap Fusion Lumos mass spectrometer (Thermo Fisher Scientific, Waltham, MA) using a 2 h gradient. Peptides were resolved using 75 *µ*m × 50 cm PepMap C18 column (Thermo Fisher Scientific, Waltham, MA).

Peptides were eluted at 300 nL/min from a 75 *µ*m × 50 cm PepMap C18 column (Thermo Scientific) using the following gradient: time = 0–4 min, 2% B isocratic; 4–8 min, 2% to 10% B; 8–83 min, 10% to 25% B; 83–97 min, 25% to 50% B; and 97–105 min, 50% to 98%. Mobile phase consisted of A, 0.1% formic acid, and mobile phase B, 0.1% formic acid in acetonitrile. The instrument was operated in the data-dependent acquisition mode in which each MS1 scan was followed by higher-energy collisional dissociation (HCD) of as many precursor ions in 2 s cycle (Top Speed method). The mass range for the MS1 done using the FTMS was 365 to 1800 m/z with resolving power set to 60,000 at 400 m/z and the automatic gain control (AGC) target set to 1,000,000 ions with a maximum fill time of 100 ms. The selected precursors were fragmented in the ion trap using an isolation window of 1.5 m/z, an AGC target value of 10,000 ions, a maximum fill time of 100 ms, a normalized collision energy of 35, and activation time of 30 ms. Dynamic exclusion was performed with a repeat count of 1, exclusion duration of 30 s, and a minimum MS ion count for triggering MS/MS set to 5,000 counts.

### APMS data analysis

MS data were converted into mgf. Database searches were done using Mascot (Matrix Science, London, UK; v.2.5.0) using the TAIR10 database (20101214, 35,386 entries) and the cRAP database (http://www.thegpm.org/cRAP/) and assuming the digestion enzyme trypsin and 2 missed cleavages. Mascot was searched with a fragment ion mass tolerance of 0.60 Da and a parent ion tolerance of 10 ppm. Oxidation of methionine and carbamidomethyl of cysteine was specified in Mascot as variable modifications. Scaffold (Proteome Software Inc., Portland, OR; v.4.8) was used to validate MS/MS-based peptide and protein identifications. Peptide identifications were accepted if they could be established at >95.0% probability by the PeptideProphet algorithm (Keller et al. 2002) with Scaffold delta-mass correction. The Scaffold Local false discovery rate (FDR) was used, and only peptide probabilities with FDR <1% were used for further analysis. Protein identifications were accepted if they could be established at >99.9% probability as assigned by the ProteinProphet algorithm (Nesvizhskii et al. 2003). Proteins that contained similar peptides and could not be differentiated based on MS/MS analysis alone were grouped to satisfy the principles of parsimony. Proteins sharing peptide evidence were grouped into clusters. Only the proteins identified with ≥ 2 unique peptides were further used in the analysis, except when proteins with only 1 peptide were identified in >1 replicate.

### Yeast 2-hybrid (Y2H) and yeast 3-hybrid assays

We used the GAL4-based Matchmaker Gold Yeast 2-Hybrid System (Clontech, Mountain View, CA) for all Y2H and Y3H assays. All transformations were performed as detailed in the Yeast Protocols Handbook (Clontech, Mountain View, CA). For Y2H, bait proteins were cloned into the pGBKT7 vector which encodes the GAL4 DNA-binding domain and then transformed into the Y2H Gold strain (Clontech, Mountain View, CA) and plated on SD/-Trp to select for positive transformants. Prey proteins were cloned into the pGADT7 vector which encodes the GAL4 activation domain, transformed into the Y187 strain (Clontech, Mountain View, CA), and plated on SD/-Leu to select for positive transformants. All matings were performed as detailed in the Yeast Protocols Handbook (Clontech, Mountain View, CA) using the 96-well plate format. Mated diploids were selected for on SD/-Leu/-Trp media. Single colonies of mated bait + prey strains were resuspended in YPDA and plated on SD/-Leu-Trp or SD/-Leu-Trp-His plates.

For Y3H, bait and linker proteins were cloned into the appropriate position of the pBridge vector (Clontech, Mountain View, CA), which encodes a GAL4 DNA-binding domain and a linker protein, transformed into the Y2H Gold strain, and plated on SD/-Trp to select for positive transformants. pBridge strains were mated with pGADT7 prey strains and plated on SD/-Trp/-Leu to select for diploids. Single colonies of mated strains were resuspended in YPDA plated on SD/-Leu-Trp or SD/-Leu-Trp-His plates.

### Luciferase reporter assays

Individual 6-d-old seedlings expressing a *CCA1::LUC* reporter grown under LD cycles at 22 °C were arrayed on 1/2x MS + 1% (*w*/*v*) sucrose plates and sprayed with 5 mM luciferin (GoldBio, Olivette, MO) prepared in 0.01% (*v*/*v*) Triton X-100 (Millipore Sigma-Aldrich, St. Louis, MO). Plants were transferred to an imaging chamber set to the appropriate free-run or entrainment program, and images were taken every 60 min with an exposure of 10 min after a 3 min delay after lights-off to diminish signal from delayed fluorescence using a Pixis 1024 CCD camera (Princeton Instruments, Trenton, NJ). Lighting conditions were 70 *µ*mol m^−2^ s^−1^ (measured with a LI-COR LI-250A, Li-COR Biosciences, Lincoln, NE), by setting wavelengths 400, 430, 450, 530, 630, and 660 nm to intensity 350 (Heliospectra LED lights). Images were processed to measure luminescence from each plant using the MetaMorph imaging software (Molecular Devices, Sunnyvale, CA). Circadian period was calculated using fast Fourier transformed nonlinear least squares (FFT-NLLS) (Plautz et al. 1997) using the Biological Rhythms Analysis Software System 3.0 (BRASS). To determine significant differences in period length between genotypes, we used an ANOVA with a Tukey's post hoc test.

For temperature entrainment experiments in Fig. 5, A to D, we used a nonramping program in which the temperature changes immediately at the transition time (i.e. after 12 h at 22 °C, the temperature will immediately drop to 20 °C). In Supplemental Fig. S13, we programed the growth chamber to run in “ramping” mode to employ a ramping temperature entrainment program which gradually oscillates between a low temperature of 16 °C at ZT16 and a high of 22 °C at ZT4. All temperature entrainment experiments were repeated independently at least twice. To determine significant differences in acrophase between genotypes at a given timepoint, we used an unpaired t-test with Welch’s correction.

### *N. benthamiana* transient transformation

Transient transformation 3-to-5-wk-old *N. benthamiana* plants was performed as in Lasierra and Prat (2018). Briefly, overnight saturated cultures of *A. tumefaciens* strain GV3101 carrying pGreenII 0800-LUC-TOC1p and pSOUP, pB7-RVE8-HFC, pB7-LNK1-HFC, pK7-COR27-VENUS-2x-StrepII-HA-6X-His-*C*-terminus (SHHc), pK7-COR28-VENUS-SHHc, pK7-VENUS-SHHc, or 35S::P19-HA (Chapman et al. 2004) were pelleted at 4000×g for 10 min, washed with a 10 mM MgCl_2_ solution, pelleted again at 4000×g for 10 min, and resuspended in 5 mL of resuspension buffer [10 mM MgSO_4_, 10 mM MES (pH 5.7), 150 *μ* M acetosyringone] for 2–4 h. Cultures were diluted with resuspension buffer to OD_600_= 0.1 for TOC1 and OD_600_= 0.4 for all other cultures. Inoculation mixtures were prepared by mixing the selected constructs together with the volume of 35S::P19-HA being varied to ensure that an equal amount of agrobacteria was added to each mixture relative to the reporter, regardless of the total number of effectors being introduced. Mixtures were inoculated into quadrants of leaves, such that 4 mixtures could be inoculated into a single leaf. Two leaves per plant were inoculated, and 4 plants were used for a total of 8 biological replicates per mixture.

### Dual-luciferase transcriptional assay

A leaf disk (11 mm) was collected from each inoculated quadrant of a 3-d postinfiltrated *N. benthamiana* leaf and placed on a 1/2x MS plate, along with a nontransfected leaf control for background. The abaxial side of the leaf discs were sprayed with a luciferin solution [5 mM luciferin, 0.04% to 0.1% TritonX-100 (*v*/*v*)], incubated for at least 20 min, and then imaged for firefly luciferase luminescence for 5 min with 2x binning using a Pixis 1024 or Pixis 2048 CCD camera (Princeton Instruments, Trenton, NJ). After imaging, the leaf discs were frozen in liquid N_2_. Tissue was ground and resuspended in 200 *μ*L of Cell Culture Lysis Reagent [100 mM potassium phosphate, pH 7.8, 1 mM EDTA, 7 mM 2-mercaptoethanol, 1% Triton X-100 (*v*/*v*), 10% glycerol (*v*/*v*), CCLR]. After freezing, grinding, and lysis, firefly luminescence was undetectable. Lysates were centrifuged at ≥18,000*×g* for 5 min, and 80 *μ*L of undiluted extract was added to a black 96-well plate and incubated for at least 10 min at room temperature with 20 *µ*L of a 1:17 dilution of a 472 mM coelenterazine luciferin (Cat. S2001, Promega) in methanol, diluted directly into CCLR before use. Renilla luminescence was measured using the above-described imaging protocol. To calculate the reporter signals, integrated intensity values were determined using MetaMorph Offline (Molecular Devices) and the average background luminescence was subtracted from the sample luminescence values. Firefly luciferase output was normalized to renilla for each sample, and reporter alone (no effector) luminescence was set to 1 to determine the fold change between conditions. Significant differences in the fold change of TOC1::LUC/35S::renilla between inoculation treatments were determined using an unpaired *t*-test with Welch’s correction.

### Densitometry analysis

Densitometry analysis was performed in FIJI (https://imagej.net/software/fiji/) on high-resolution (600 dpi), grayscale images of western blots captured with the same exposure time. Mean gray value was measured from regions of interest (ROIs) of equal area for each protein band and for background regions as well as for loading controls (Ponceau S stain) and loading control background regions. Inverted pixel density of background regions was subtracted from the inverted pixel density of protein bands and loading controls to generate the net pixel density value. To calculate normalized abundance, the ratio of the net protein band value over the net loading control value was taken. The max intensity value for each biorep was set to 100 for each genotype. Significant differences in protein abundance between genotypes at a given timepoint were determined using Welch's *t*-test.

### Reverse-transcription quantitative PCR (RT-qPCR)

Seedlings were gas sterilized and grown on 1/2x MS + 1% sucrose plates with Whatman filter paper under 12 h light:12 h dark, 22 °C conditions. On Day 7 of growth at ZT10, plates were transferred to a different chamber set to either 22 °C or 4 °C for 2 h. Tissue was collected at ZT12. Total RNA was extracted from powdered tissue using the RNeasy Plant Mini kit (Qiagen, Hilden, Germany). One *µ*g of total RNA was used as the template to synthesize cDNA using the iScript RT-PCR kit (Bio-Rad, Carlsbad, CA). RT-qPCR was performed with the SYBR Green I nucleic acid gel stain (Sigma-Aldrich) using a QuantStudio 5 Real-Time PCR System (Thermo Fisher). PCR was set up as follows: 3 min at 95 °C, followed by 40 cycles of 10 s at 95 °C, 10 s at 55 °C, and 20 s at 72 °C. A melting curve analysis was conducted right after all PCR cycles are done. APA1 (At1g11910), expression of which remain stable during the diurnal cycle, was used as the normalization control. Primers for RT-qPCR are listed in Supplemental Table S2.

### Protein alignment of TCF1 (AT3G55580) and RCC1L (AT3G53830)

Protein sequences of TCF1 (AT3G55580) and RRC1L (AT3G53830) were aligned using the needle algorithm using the EBLOSUM62 matrix, a gap penalty of 10.0, and an extend penalty of 0.5.

### Accession numbers

The mass spectrometry proteomics data have been deposited to the ProteomeXchange Consortium via the PRIDE (Perez-Riverol et al. 2022) partner repository with the data set identifier PXD034089 and 10.6019/PXD034089.

## Supplementary Material

kiad210_Supplementary_Data

## Supplemental data

The following materials are available in the online version of this article.

**Supplemental Data Set 1**. Complete data sets for LNK1-HFC, LNK2-HFC, RVE8-HFC, YFP-COR27, and GFP-COR28 APMS experiments.

**Supplemental Figure S1**. Protein abundance patterns of affinity-tagged lines (biorep 2).

**Supplemental Figure S2**. mRNA expression patterns of *RVE8*, *LNK1*, or *LNK2* under photocycles (12 h light:12 h dark).

**Supplemental Figure S3**. Protein alignment of TCF1 (AT3G55580) and RCC1L (AT3G53830).

**Supplemental Figure S4**. Comparison of HFC-tagged protein abundance with *COR27/28*, *COP1*, and *SPA1* mRNA expression profiles.

**Supplemental Figure S5**. COR27/28 do not interact with RVE8 or LNK1 in a binary Y2H system.

**Supplemental Figure S6**. Full-length LNK1 autoactivates in yeast when paired with a DBD-containing protein.

**Supplemental Figure S7**. RVE8-HFC protein abundance patterns are regulated by the 26S proteasome.

**Supplemental Figure S8**. Additional bioreps of RVE8-HFC *cor27-2 cor28-*2 24 h collections.

**Supplemental Figure S9**. The RVEs and LNKs are important for cold induction of *COR27/28*.

**Supplemental Figure S10**. Rayleigh plots of *lnk* mutant acrophase under 22 °C:20 °C temperature entrainment.

**Supplemental Figure S11**. Rayleigh plots of *lnk* mutant acrophase under 22 °C:18 °C temperature entrainment.

**Supplemental Figure S12**. Rayleigh plots of *lnk* mutant acrophase under photoentrainment.

**Supplemental Figure S13**. *lnk* mutants are also impaired in temperature entrainment under ramping temperature cycles.

**Supplemental Figure S14**. *lnk3/4* mutants are not impaired in temperature entrainment. *RCC1L* (*AT3G53830*) expression is downregulated by cold treatment.

**Supplemental Table S2**. Oligonucleotides used in this study.
