# Supplementary material for: COLD REGULATED GENE 27 and 28 antagonize the transcriptional activity of the RVE8/LNK1/LNK2 circadian complex
Source: Plant Physiol. 2023 Apr 5;192(3):2436–56. doi: 10.1093/plphys/kiad210 (PMC10315267; doi:10.1093/plphys/kiad210)
Supplement: kiad210_Supplementary_Data [file kiad210_supplementary_data.zip › Supplemental Figures Combined.pdf]

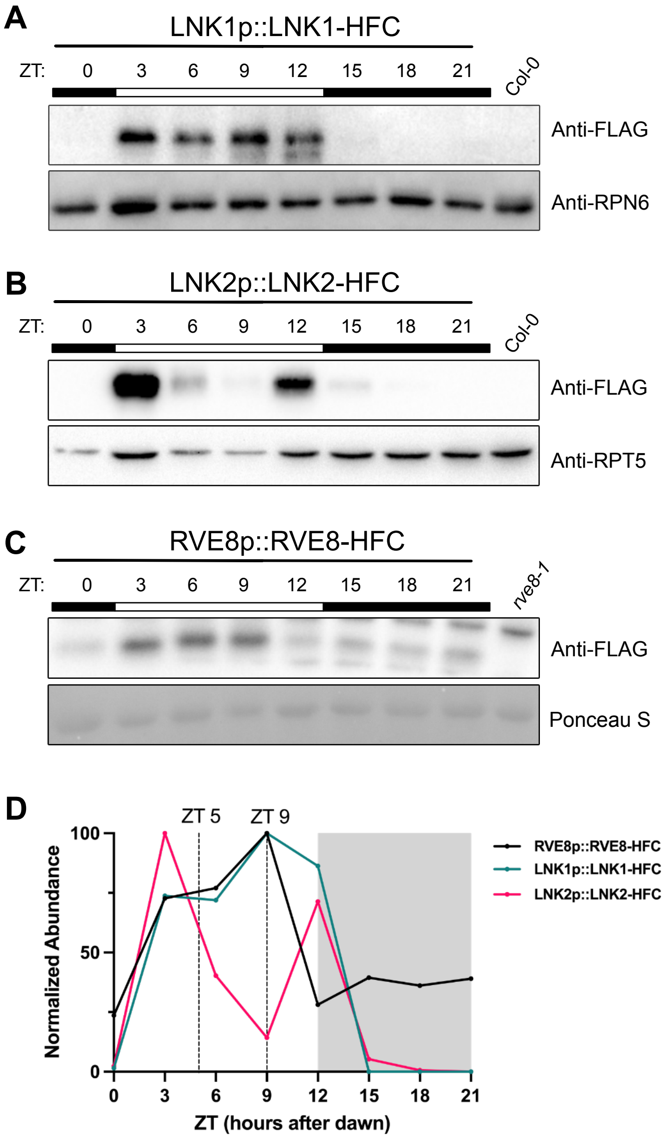

Supplemental Figure S1. Protein abundance patterns of affinity-tagged lines (biorep 2). (A-C) Time course Western blots showing cyclic protein abundance patterns of 10-day-old affinity tagged lines under 12 hr light: 12 hr dark 22 °C conditions. Affinity tagged lines are detected with anti-FLAG antibody. RPT5, RPN6, or Ponceau S staining was used to show loading. Col-0 *CCA1::LUC* (Col-0) or *rve8-1* CCR2::LUC (*rve8-1*) were used as negative controls. White and black bars indicate lights-on and lights-off, respectively. D) 24-hour protein expression patterns of affinity tagged lines normalized to Ponceau S, RPN6, or RPT5 quantified by densitometry of Western blots shown in A-C. Vertical dotted lines indicate time of tissue collection for APMS. White and grey shading indicates lights-on and lights-off, respectively. This is the second biorep, with biorep 1 shown in Figure 1. ZT= Zeitgeber Time.

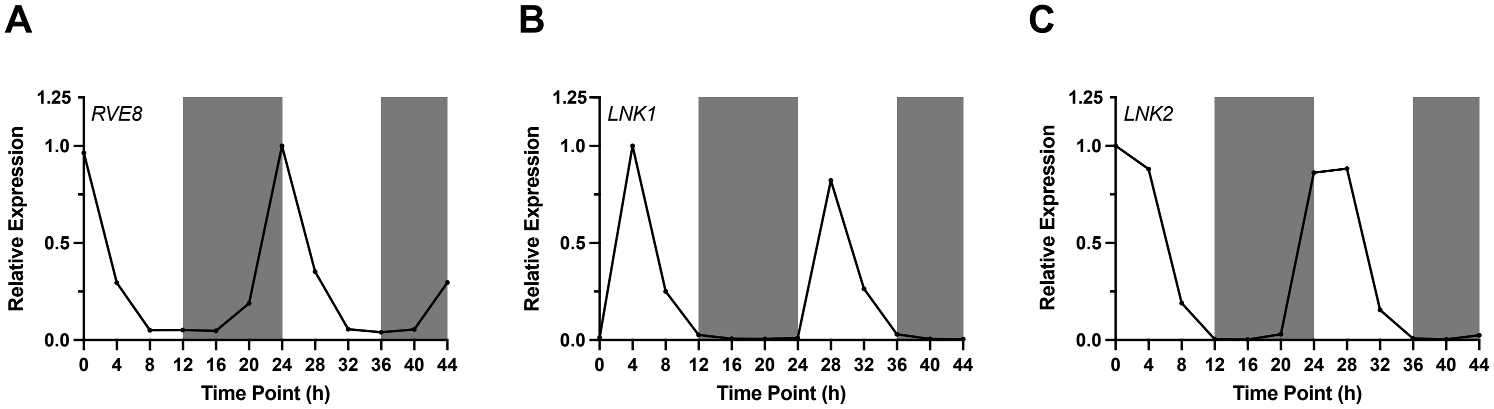

Supplemental Figure S2. mRNA expression patterns of *RVE8*, *LNK1*, or *LNK2* under photocycles (12 hr light: 12 hr dark). White and dark grey shading indicates lights-on and lights-off, respectively. Microarray data from diurnal.mocklerlab.com.

```
TCF1                1 MNGE--GKLETGGTSAAATPKEEKEEEEVVSQRVVYMWGYLPGASPQRSP     48
                      |||.  ||:..|...|.                ||||.||||||:.::||
RCC1L               1 MNGNIVGKVPIGECKAT----------------VVYMSGYLPGAASEKSP     34

TCF1               49 LMSPVEVKIPPAVE--SSWKDVSGGGCGFAMATAESGKLITWGSTDDLGQ     96
                      ::|||.|::..||.  .|||||.|||||||||.:|.|||||||||||.||
RCC1L              35 ILSPVPVRLSAAVHGGDSWKDVCGGGCGFAMAISEKGKLITWGSTDDEGQ     84

TCF1               97 SYVTSGKHGETPEPFPLPPEVCVQKAEAGWAHCVAVTENQQVYTWGWREC    146
                      |||.||||||||||||||.|..|.:|.:|||||..|||..:.:||||:||
RCC1L              85 SYVASGKHGETPEPFPLPTEAPVVQASSGWAHCAVVTETGEAFTWGWKEC    134

TCF1              147 IPTGRVFGQVDGDSCERNISFSTEQVSSSSQGKKSSGGTSSQVEG-RGGG    195
                      ||:        .|...:..|.|:||||.:|||..::.||:.|.|. :.|.
RCC1L             135 IPS--------KDPVGKQQSGSSEQVSPASQGSNAASGTTLQNENQKVGE    176

TCF1              196 EPTKKRRISPSKQAAENSSQSDNIDLSALPCLVSLAPGVRIVSVAAGGRH    245
                      |..|:||:|.:|...|..:...:. .:..|.|||:..||||.|||.||||
RCC1L             177 ESVKRRRVSTAKDETEGHTSGGDF-FATTPSLVSVGLGVRITSVATGGRH    225

TCF1              246 TLALSDIGQVWGWGYGGEGQLGLGSRVRLVSSPHPIPCIEPSSYGKATS-    294
                      ||||||:||:||||||||||||||||:::|||||.|||:|....||..|
RCC1L             226 TLALSDLGQIWGWGYGGEGQLGLGSRIKMVSSPHLIPCLESIGSGKERSF    275

TCF1              295 ---SGVNMSSVVQCGRVLGSYVKKIACGGRHSAVITDTGALLTFGWGLYG    341
                         .|...::..|..|..|.|:|.|:|||||||.|||.|.|:||||||||
RCC1L             276 ILHQGGTTTTSAQASREPGQYIKAISCGGRHSAAITDAGGLITFGWGLYG    325

TCF1              342 QCGQGSTDDELSPTCVSSLLGIRIEEVAAGLWHTTCASSDGDVYAFGGNQ    391
                      |||.|:|:|:|.|..||.:..:|:|.||||||||.|.||||.||||||||
RCC1L             326 QCGHGNTNDQLRPMAVSEVKSVRMESVAAGLWHTICISSDGKVYAFGGNQ    375

TCF1              392 FGQLGTGCDQAETLPKLLEAPNLENVNVKTISCGARHTA----VITDEGR    437
                      |||||||.|.||.||:||:..|||..:.|.:||||||:|    |:|...
RCC1L             376 FGQLGTGTDHAEILPRLLDGQNLEGKHAKAVSCGARHSAVLAVVLTRRN-    424

TCF1              438 VFCWGWNKYGQLGIGDVIDRNAPAEVRIKDCFPKNIACGWWHTLLLGQPT    487

RCC1L             425 --------------------------------------------------    424

TCF1              488 L    488

RCC1L             425 -    424
```

Supplemental Figure S3. Protein alignment of TCF1 (AT3G55580) and RCC1L (AT3G53830). Protein sequences were aligned using the needle algorithm using the EBLOSUM62 matrix, a gap penalty of 10.0, and an extend penalty of 0.5. Sequences share 49.7% identity.

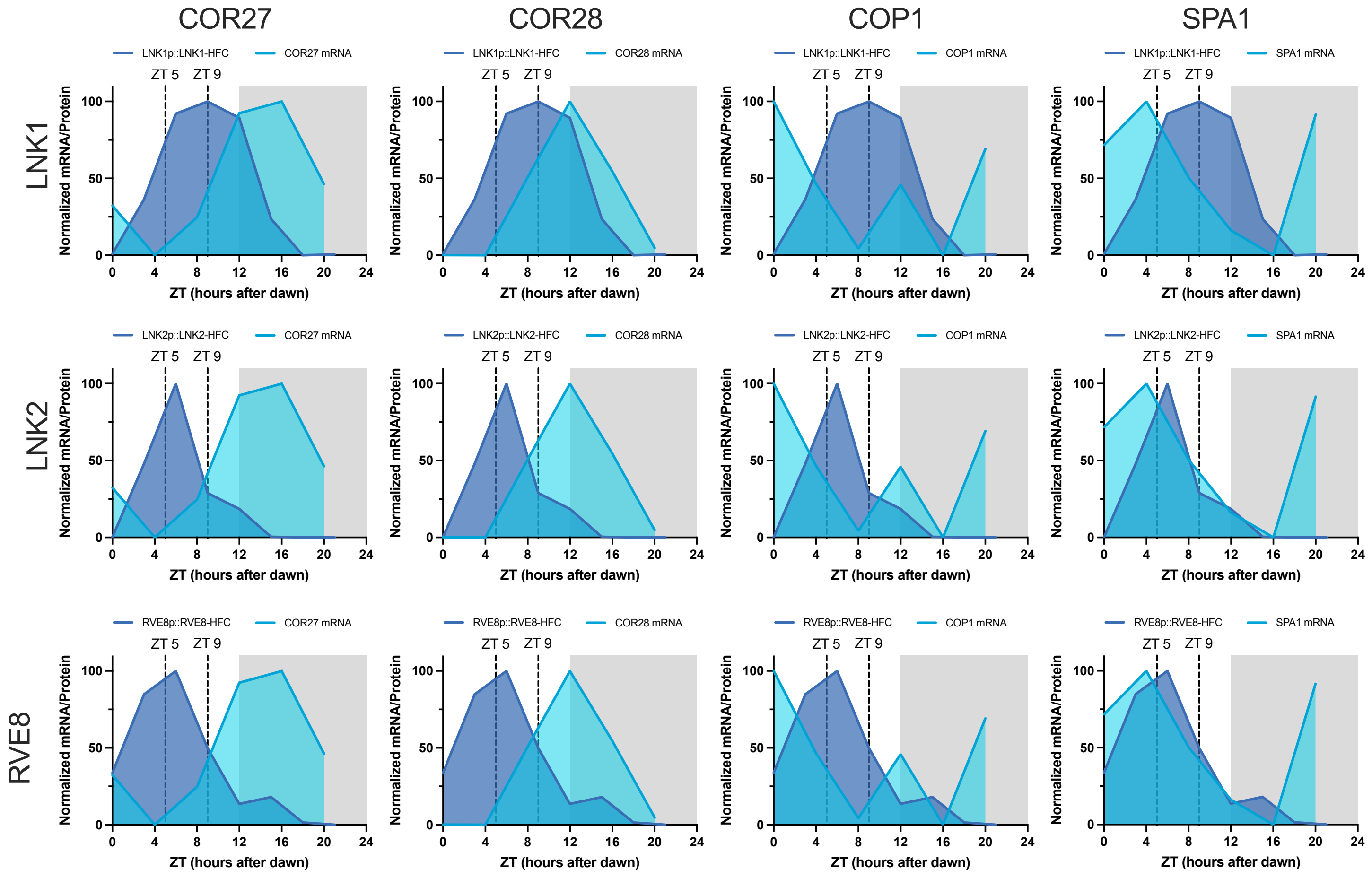

Supplemental Figure S4. Comparison of HFC-tagged protein abundance with *COR27/28*, *COP1*, and *SPA1* mRNA expression profiles. 24-hour (12 hr light: 12 hr dark, 22 °C (LDHH)) protein abundance (dark blue) is quantified from Western blots shown in Figure 1D-F. LDHH mRNA data from diurnal.mocklerlab.com (light blue) is overlayed. Vertical dotted lines show the time of day when tissue was collected for APMS. White and grey shading indicated lights-on and lights-off, respectively. ZT= Zeitgeber Time.

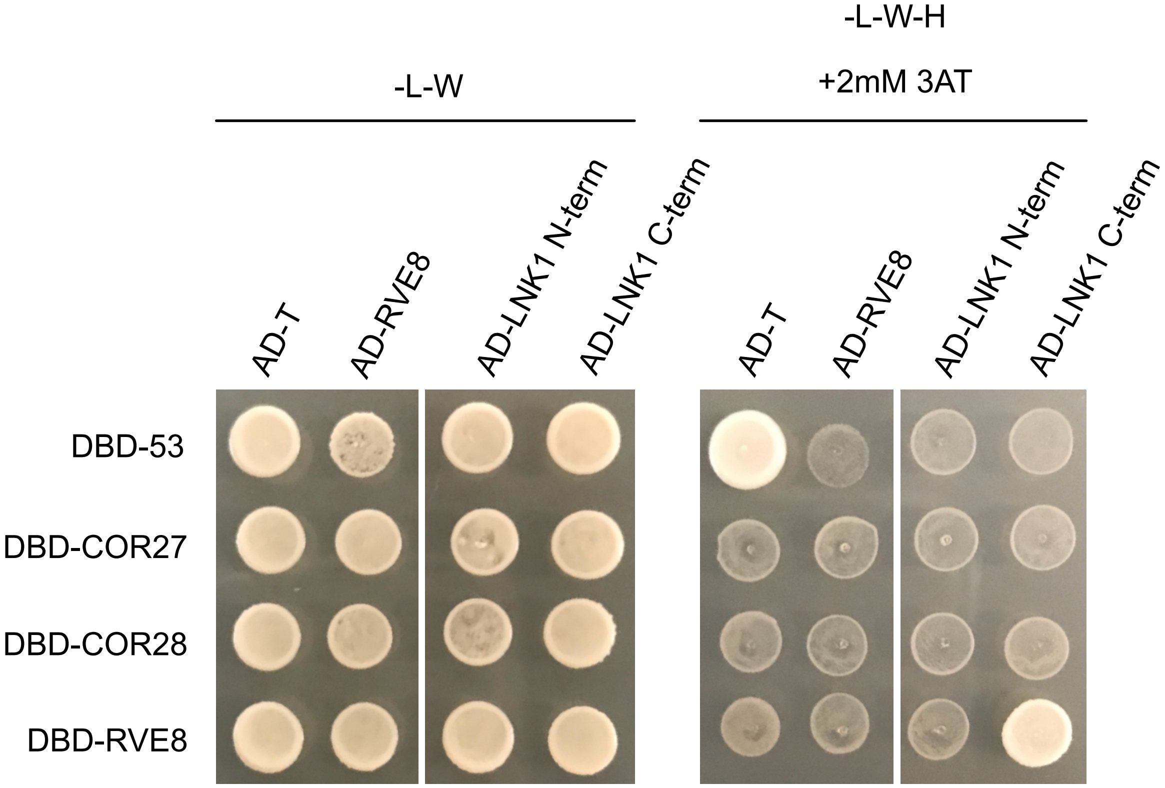

Supplemental Figure S5. COR27/28 do not interact with RVE8 or LNK1 in a binary Y2H system Yeast strains Y2H Gold or Y187 expressing pGBKT7 (Gal4-DBD) or pGADT7 (Gal4-AD), respectively, were mated and plated onto selective media. Successful matings were able to grow on -Leucine/-Tryptophan media (-L-W) while positive interactors can grow on -Leucine/-Tryptophan/-Histidine + 2mM 3-amino-1,2,4-triazole (3AT) (-L-W-H +3AT). Only the positive controls DBD-53 (p53) + AD-T (large T-antigen protein) and DBD-RVE8 + AD-LNK1 C-term show an interaction.

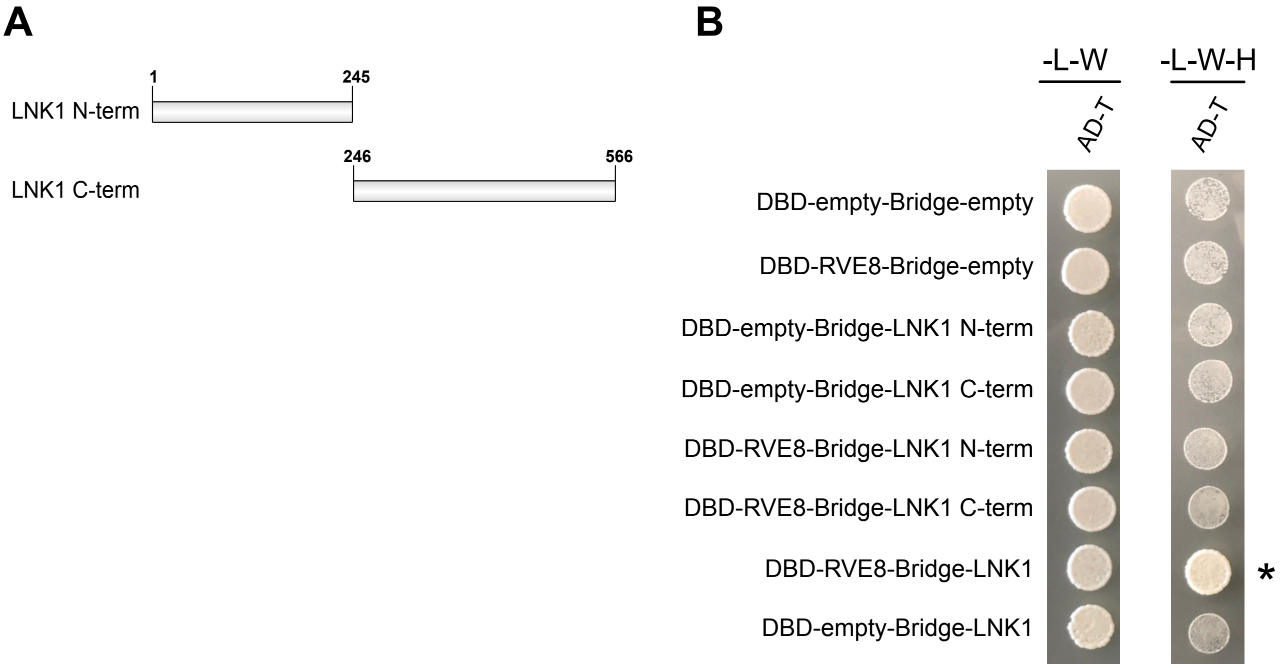

Supplemental Figure S6. Full-length LNK1 auto-activates in yeast when paired with a DBD-containing protein. A) Protein diagrams showing N- and C-terminal truncations of LNK1 used in yeast assays. B) Yeast strains Y2H Gold or Y187 expressing pBridge (Gal4-DBD and a Bridge protein) or pGADT7 (Gal4-AD), respectively, were mated and plated onto selective media. Successful matings were able to grow on -Leucine/-Tryptophan media (-L-W). Full length LNK1 (bridge protein, no AD domain) paired with the transcription factor RVE8 (*) can aberrantly activate the expression of the histidine biosynthesis reporter, allowing it to grow on -Leucine/-Tryptophan/-Histidine (-L-W-H) when paired with the negative control large T-antigen protein (T). LNK1 N- and C-terminal truncations do not autoactivate.

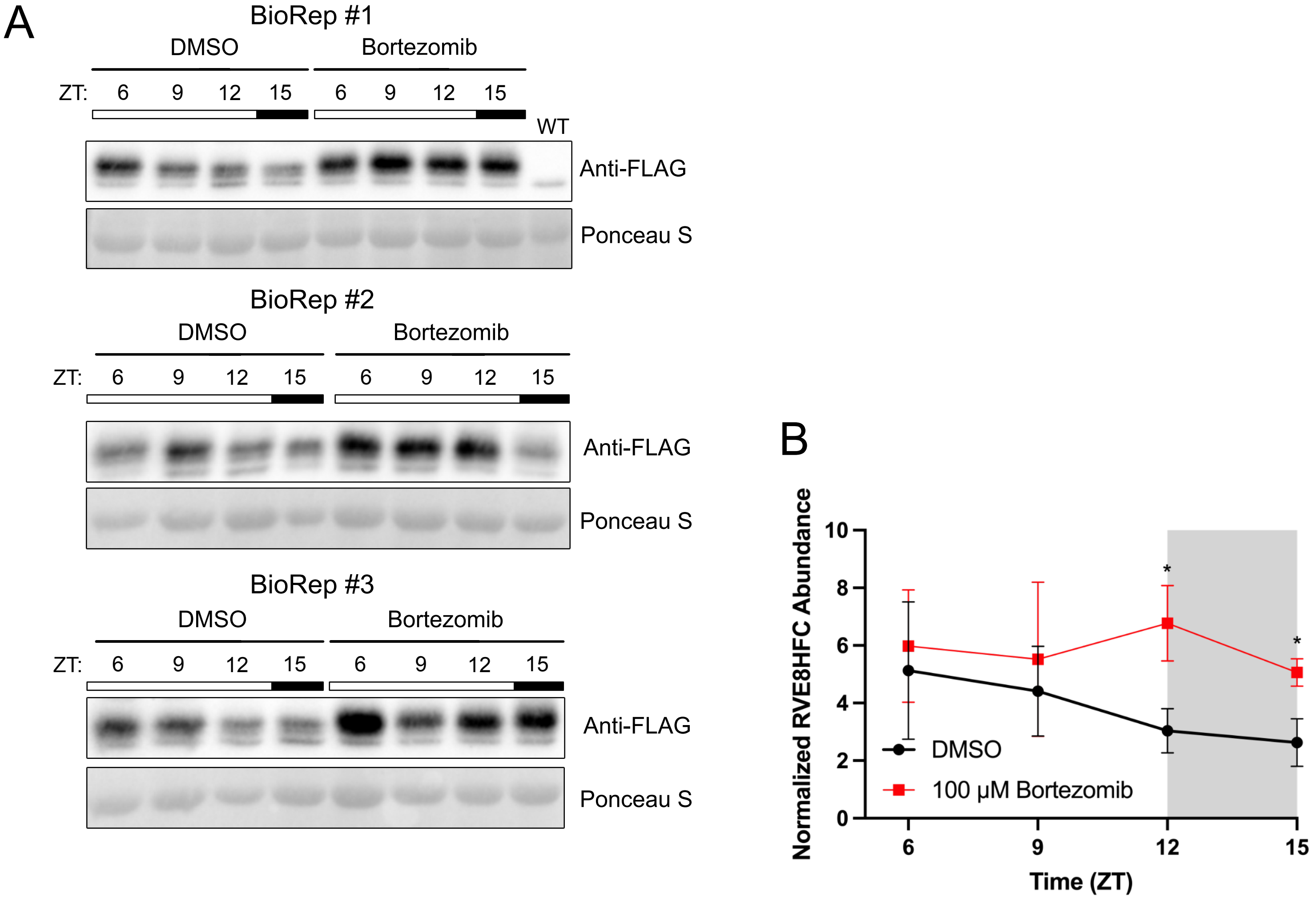

Supplemental Figure S7. RVE8-HFC protein abundance patterns are regulated by the 26S proteasome. (A) Western blots showing protein expression patterns of RVE8-HFC plants treated with DMSO or 100 µM bortezomib in three independent biological replicates. At ZT5, 12-day-old seedlings growing under 12 hr light: 12 hr dark, 22 °C conditions were immersed in 1/2X MS media containing either 100 µM bortezomib or DMSO. Tissue was collected every 3 hours starting at ZT6. RVE8-HFC was detected with anti-FLAG and Ponceau S staining was used to show loading. (B) Densitometry quantification of RVE8-HFC abundance in (A) normalized to Ponceau S. Points represent the average normalized RVE8-HFC abundance from the 3 independent bioreps shown in (A). Asterisks indicate significant differences between genotypes based on Welch's t-test (* $p<0.05$). Error bars = SD. White and grey shading indicate lights-on and lights-off, respectively. ZT= Zeitgeber Time.

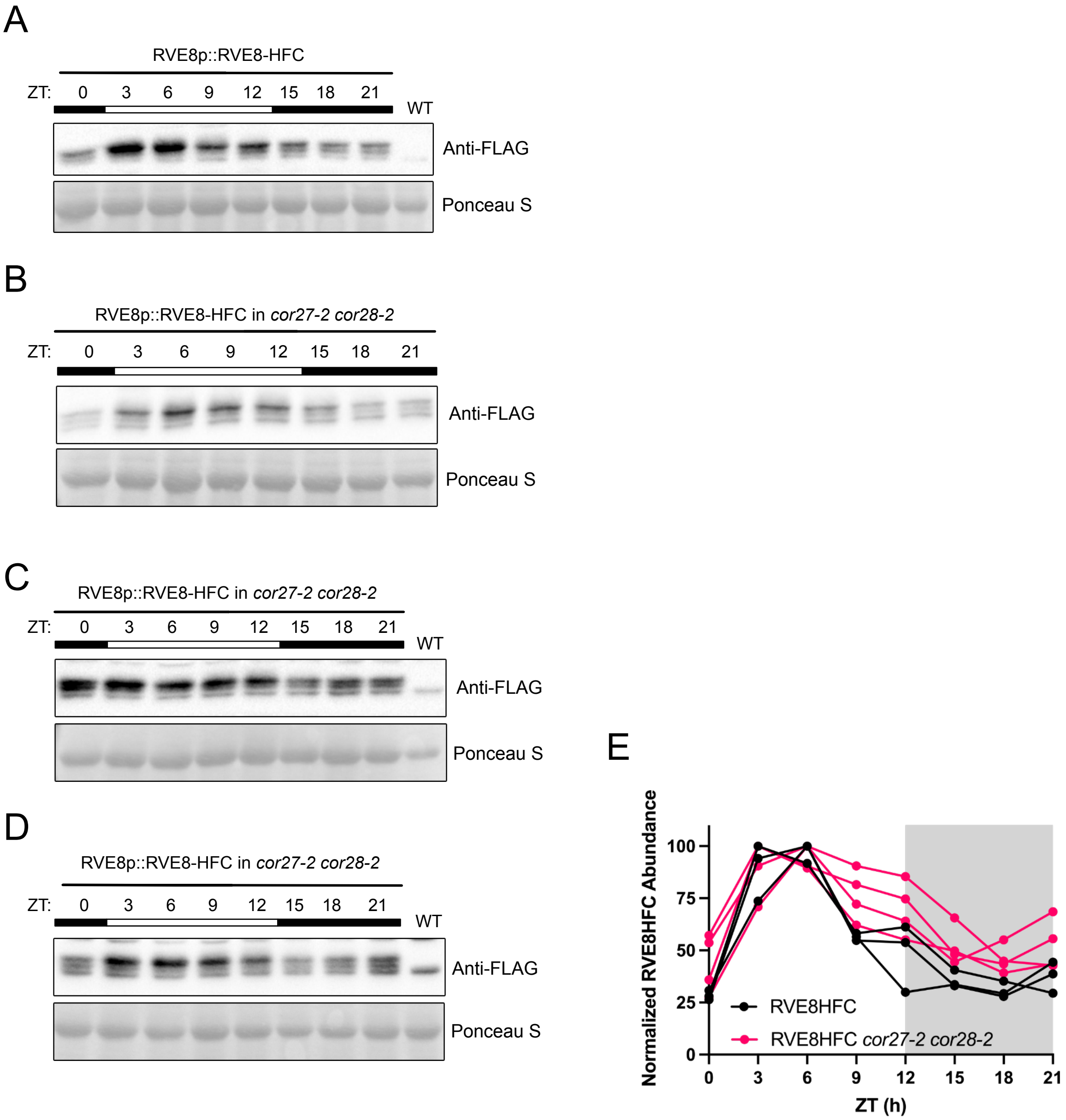

Supplemental Figure S8. Additional bioreps of RVE8-HFC *cor27-2 cor28-2* 24-hour collections. (A-D) 24-hour protein expression patterns of RVE8-HFC in wild type (A) or *cor27-2 cor28-2* (B-D) backgrounds analyzed by Western blot. The other two bioreps for RVE8p::RVE8-HFC are shown in Fig. 1F and Fig. 4A. The other biorep for RVE8-HFC *cor27-2 cor28-2* is shown in Fig. 4B. Tissue was collected every 3 hours from 12-day-old plants grown under 12 hr light: 12 hr dark 22 °C conditions. Anti-FLAG antibody was used to detect RVE8-HFC and Ponceau S staining was used to show loading. White and black bars indicate lights-on and lights-off, respectively. Col-0 *CCR2::LUC* (WT) was used as the negative control. (E) Densitometry quantification of Western blots shown in A-D as well as in the bioreps shown in Figs. 1F and 4A-B. Points represent the individual normalized RVE8-HFC abundance from 3 (WT) or 4 (*cor27-2 cor28-2*) independent bioreps. ZT= Zeitgeber Time.

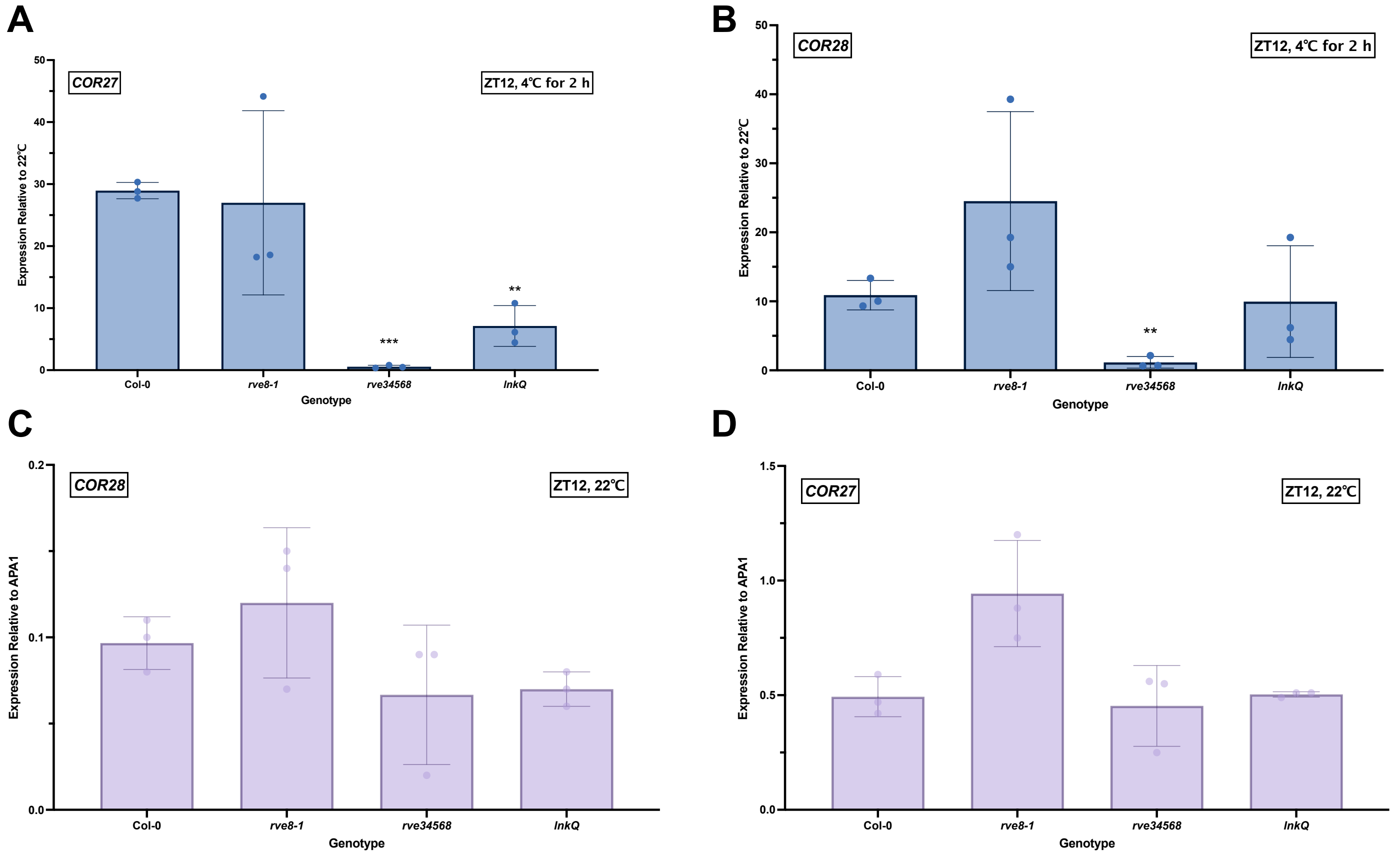

Supplemental Figure S9. The RVEs and LNKs are important for cold induction of *COR27/28*. Seedlings were grown on 1/2X MS + 1% sucrose for seven days under 12 hr light; 12 hr dark 22 °C conditions and then transferred at ZT10 to either 22 °C or 4 °C for two hours and tissue was collected at ZT12. (A-B) show the induction of *COR27/28* expression at 4 °C compared to 22 °C . Figures (C-D) show *COR27/28* expression levels at 22 °C . Expression was normalized to the endogenous control gene APA1. Bars show average expression with error bars = SD from 3 independent bioreps (points) for each genotype. Asterisks indicate significant differences as determined by Welch's t-test (** $p<0.01$, *** $P<0.001$).

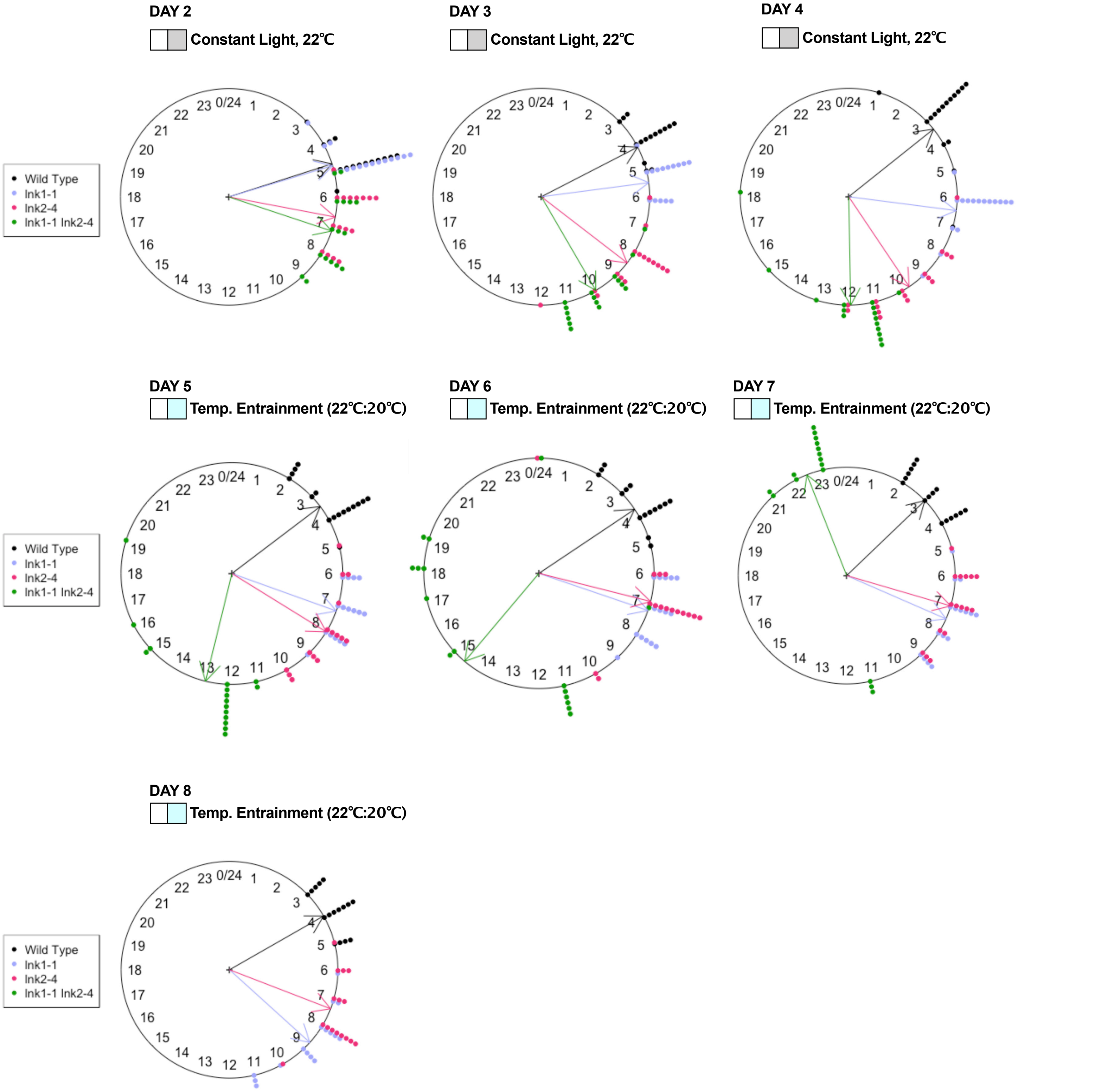

Supplemental Figure S10. Rayleigh plots of *lnk* mutant acrophase under 22 °C: 20 °C temperature entrainment. Data is from the same experiment shown in main Figure 5 A-B. Luminescence from 7-day-old plants entrained under 12 hr light: 12 hr dark, 22 °C conditions expressing a *CCA1::LUC* reporter was imaged for 3 days in continuous light and temperature (22 °C) before the chamber was switched to a block temperature entrainment program that consisted of a day temperature of 22 °C and nighttime temperature of 20 °C. Points represent the peak expression time (acrophase) of the *CCA1::LUC* reporter in individual plants for a given day in the experiment. Arrows point to the average acrophase for a given genotype.

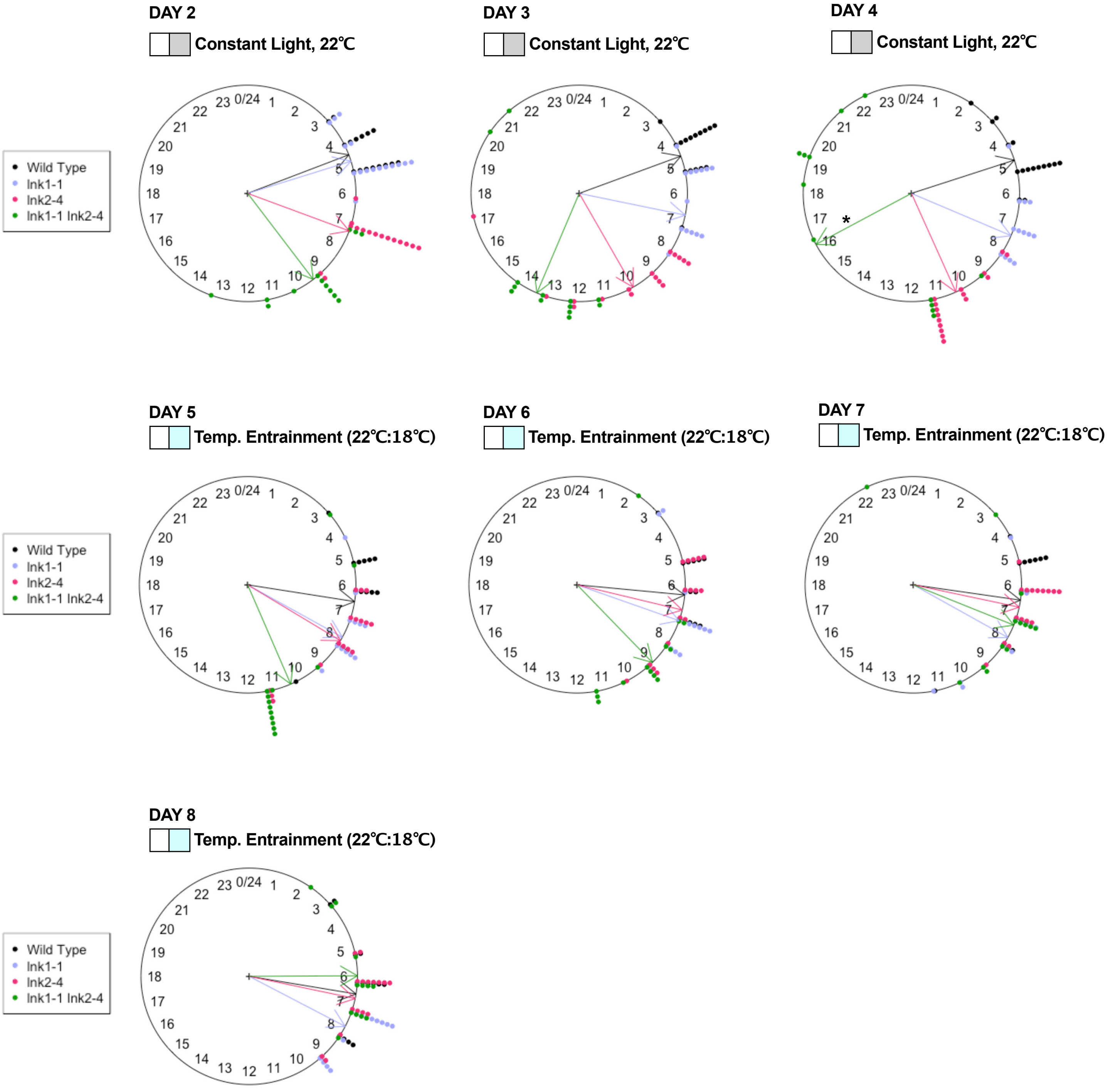

Supplemental Figure S11. Rayleigh plots of *lnk* mutant acrophase under 22 ℃: 18 ℃ temperature entrainment. Data is from the same experiment shown in main Figure 5 C-D. Luminescence from 7-day-old plants entrained under 12 hr light: 12 hr dark, 22 °C conditions expressing a *CCA1::LUC* reporter was imaged for 3 days in continuous light and temperature (22 °C) before the chamber was switched to a block temperature entrainment program that consisted of a day temperature of 22 °C and nighttime temperature of 18 °C. Points represent the peak expression time (acrophase) of the *CCA1::LUC* reporter in individual plants for a given day in the experiment. Arrows point to the average acrophase for a given genotype. Asterisk indicates a given genotype significantly deviates from a Rayleigh test for uniformity.

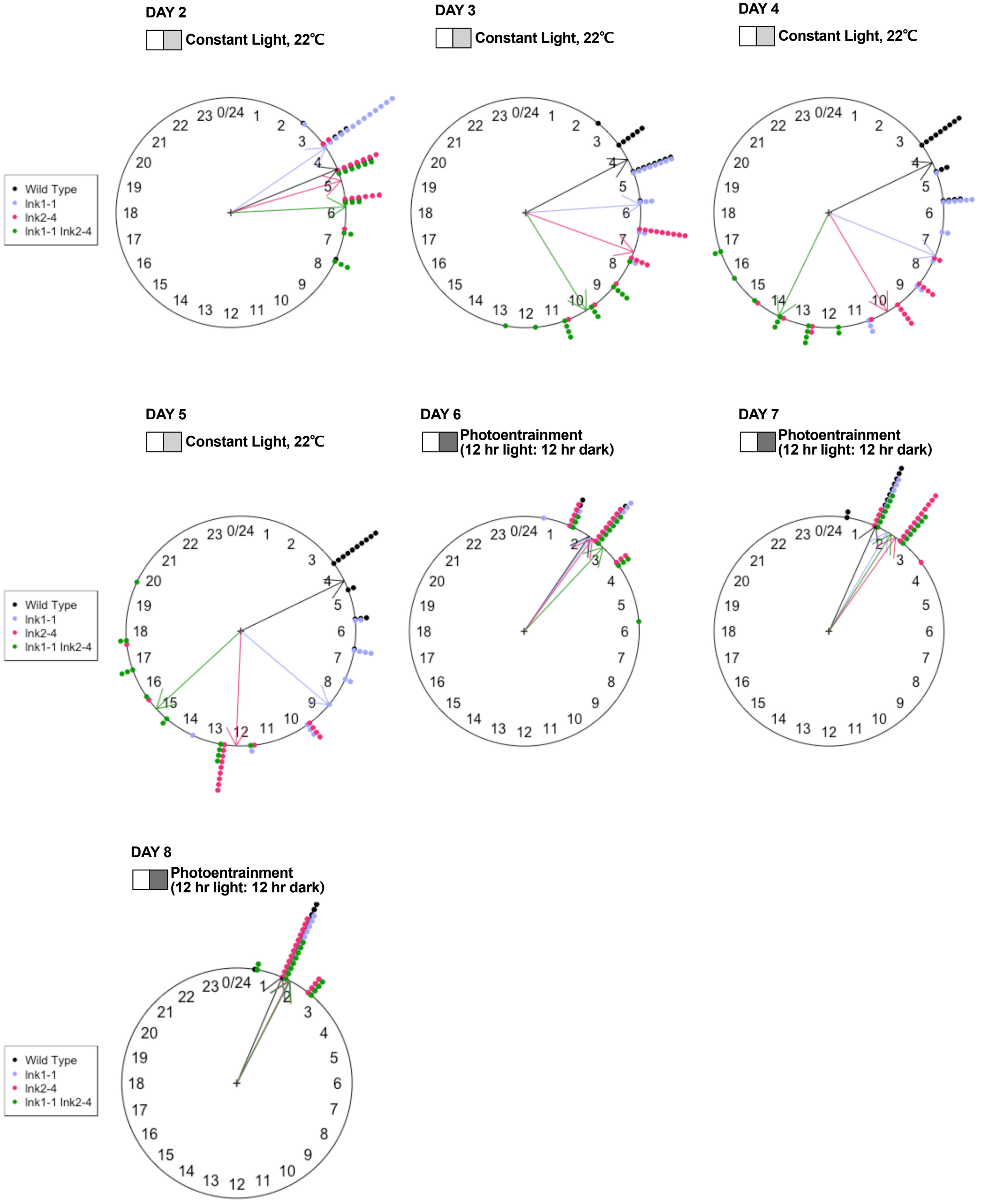

Supplemental Figure S12. Rayleigh plots of *lnk* mutant acrophase under photoentrainment. Data is from the same experiment shown in main Figure 5 E-F. Luminescence from 7-day-old plants entrained under 12 hr light: 12 hr dark, 22 °C conditions expressing a *CCA1::LUC* reporter was imaged for 3 days in continuous light and temperature (22 °C) before the chamber was switched to a photoentrainment program consisting of 12 hours light and 12 hours dark. Points represent the peak expression time (acrophase) of the *CCA1::LUC* reporter in individual plants for a given day in the experiment. Arrows point to the average acrophase for a given genotype.

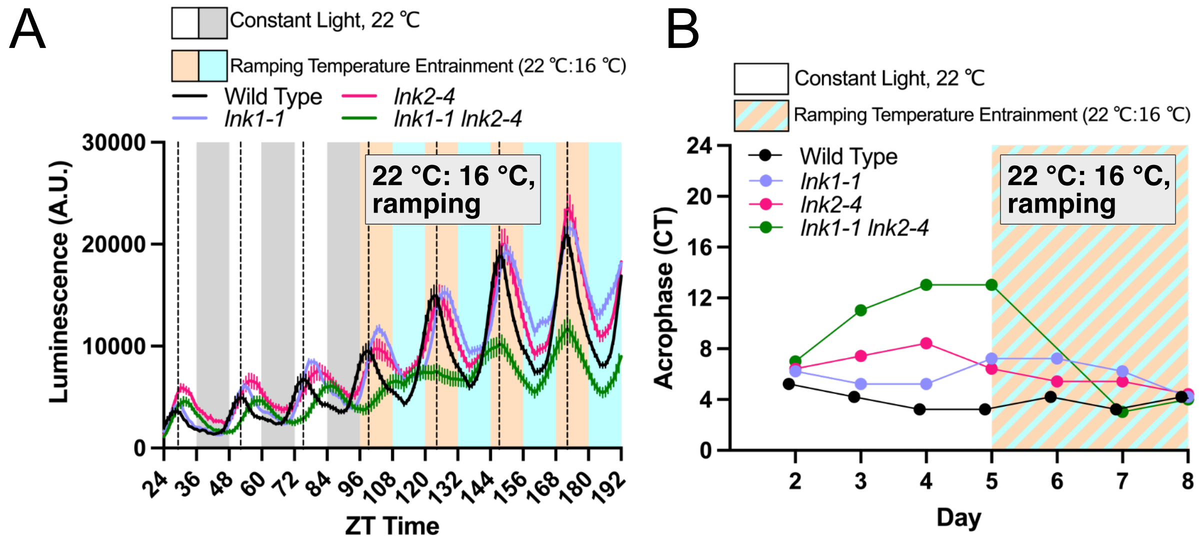

Supplemental Figure S13. *lnk* mutants are also impaired in temperature entrainment under ramping temperature cycles. (A) Luminescence from 7-day-old plants entrained under 12 hr light: 12 hr dark, 22 °C conditions expressing a CCA1p::LUC reporter was imaged for at least 3 days in continuous light and temperature (22 °C) before the chamber was switched to a ramping temperature entrainment program that gradually oscillated between a low temperature of 16 °C at ZT16 and a high of 22 °C at ZT4. Lines represent the average luminescence from n=16 seedlings with errors bars = SEM. Vertical dotted lines correspond to the peak expression time (acrophase) of the CCA1p::LUC reporter in wild type plants. (B) Acrophase, or time of peak reporter expression, is plotted for each genotype for each day of imaging in constant light and the temperature entrainment condition. Each point represents the acrophase of the averaged luminescence trace shown in (A). CT = Circadian Time. A.U. = Arbitrary Units. ZT= Zeitgeber Time.

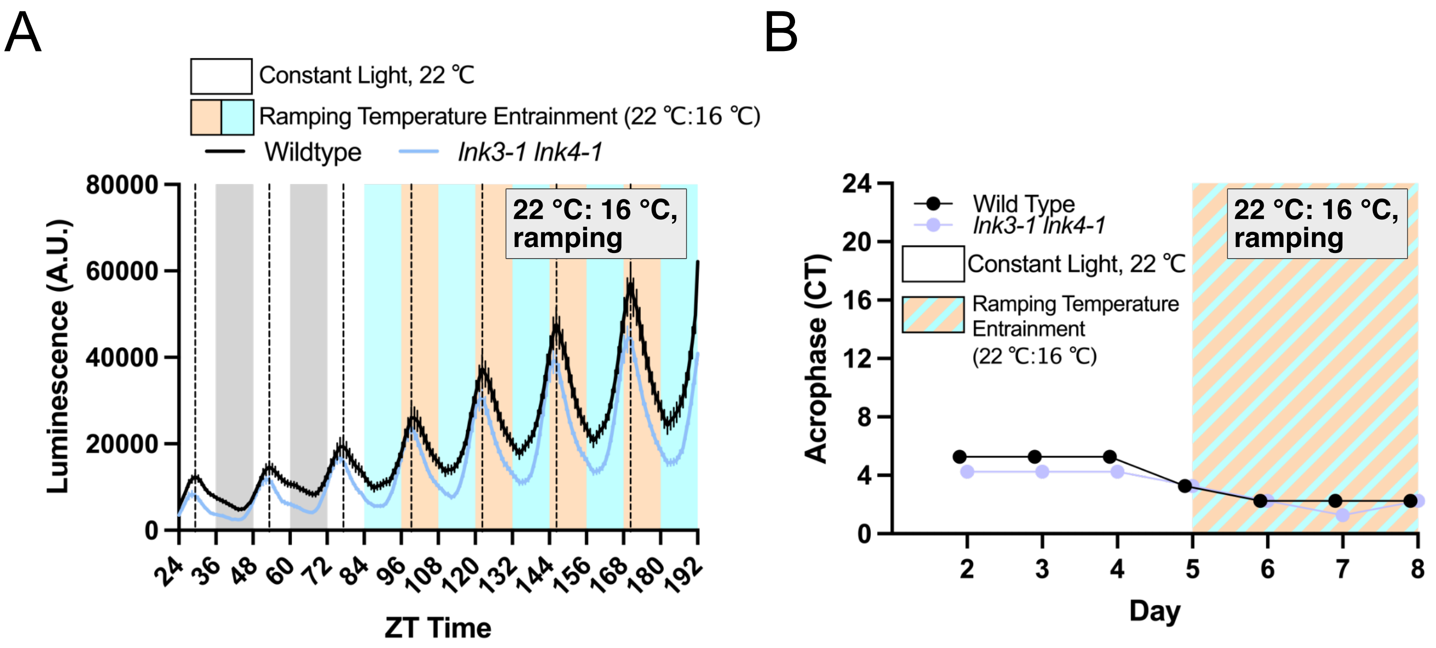

Supplemental Figure S14. ***lnk3/4*** **mutants are not impaired in temperature entrainment.** (A) Luminescence from 7-day-old plants expressing a *CCA1::LUC* reporter were grown for at least 3 days in continuous light and temperature (22 °C) before the chamber was switched to a ramping temperature entrainment program that gradually oscillates between a low temperature of 16 °C at ZT16 and a high of 22 °C at ZT4. Lines represent the average luminescence from n=16 seedlings with errors bars = SEM. Vertical dotted lines correspond to the peak expression time of the *CCA1::LUC* reporter in wild type plants. (B) Acrophase, or time of peak reporter expression, is plotted for each genotype for each day of imaging in constant light and the temperature entrainment condition. Each point represents the acrophase of the averaged luminescence trace shown on the right. CT = Circadian Time. A.U. = Arbitrary Units. ZT=Zeitgeber Time.
